# Enhancing mitochondrial proteolysis alleviates alpha-synuclein-mediated cellular toxicity

**DOI:** 10.1038/s41531-024-00733-y

**Published:** 2024-06-21

**Authors:** Xi Zhang, Linhao Ruan, Hu Wang, Jin Zhu, Taibo Li, Gordon Sun, Yi Dong, Yuhao Wang, Gil Berreby, Ashley Shay, Rong Chen, Sreekumar Ramachandran, Valina L. Dawson, Ted M. Dawson, Rong Li

**Affiliations:** 1 Center for Cell Dynamics, Department of Cell Biology, Johns Hopkins University School of Medicine, Baltimore, MD 21205 USA; 2 Diana Helis Henry Medical Research Foundation, New Orleans, LA 70130-2685 USA; 3 Neuroregeneration and Stem Cell Programs, Institute for Cell Engineering, Johns Hopkins University School of Medicine, Baltimore, MD 21205 USA; 4 Department of Neurology, Johns Hopkins University School of Medicine, Baltimore, MD 21205 USA; 5 Mechanobiology Institute, National University of Singapore, Singapore, 117411 Singapore; 6 Department of Biomedical Engineering, Johns Hopkins University School of Medicine, Baltimore, MD 21218 USA; 7 Department of Chemical and Biomolecular Engineering, Whiting School of Engineering, Johns Hopkins University, Baltimore, MD 21218 USA; 8 Solomon H. Snyder Department of Neuroscience, Johns Hopkins University School of Medicine, Baltimore, MD 21205 USA; 9 Department of Pharmacology and Molecular Sciences, Johns Hopkins University School of Medicine, Baltimore, MD 21205 USA; 10 Department of Biological Sciences, National University of Singapore, Singapore, 117411 Singapore

**Keywords:** Parkinson's disease, Cell biology

## Abstract

Parkinson’s disease (PD) is a progressive neurodegenerative disease characterized by mitochondrial dysfunction and accumulation of alpha-synuclein (α-Syn)-containing protein aggregates known as Lewy bodies (LB). Here, we investigated the entry of α-Syn into mitochondria to cause mitochondrial dysfunction and loss of cellular fitness in vivo. We show that α-Syn expressed in yeast and human cells is constitutively imported into mitochondria. In a transgenic mouse model, the level of endogenous α-Syn accumulation in mitochondria of dopaminergic neurons and microglia increases with age. The imported α-Syn is degraded by conserved mitochondrial proteases, most notably NLN and PITRM1 (Prd1 and Cym1 in yeast, respectively). α-Syn in the mitochondrial matrix that is not degraded interacts with respiratory chain complexes, leading to loss of mitochondrial DNA (mtDNA), mitochondrial membrane potential and cellular fitness decline. Importantly, enhancing mitochondrial proteolysis by increasing levels of specific proteases alleviated these defects in yeast, human cells, and a PD model of mouse primary neurons. Together, our results provide a direct link between α-synuclein-mediated cellular toxicity and its import into mitochondria and reveal potential therapeutic targets for the treatment of α-synucleinopathies.

## Introduction

Alpha-synuclein is a presynaptic protein prominently implicated in Parkinson’s disease (PD), the second largest neurodegenerative disorder characterized by symptoms of motor dysfunction that result from progressive loss of dopaminergic (DA) neurons in the substantia nigra^1^. A hallmark of PD is the formation of α-Syn-containing neuronal aggregates known as Lewy bodies and Lewy neurites, in an age-dependent manner^2–4^. α-Syn is a 140 amino acids (aa) polypeptide that contains three domains^5^: N-terminal lipid-binding α-helix (residues 1–60), amyloid-binding central domain (NAC, residues 61–95)^6^, and C-terminal acidic tail (residues 95–140). Mutations in or elevated dosage of the SNCA gene encoding α-Syn causes familial PD^7^. In animal and cell models, overexpression of α-Syn is sufficient to cause cellular toxicity and PD-like pathology and symptoms^8^. However, the mechanism underlying α-Syn toxicity remains enigmatic. Equally unclear is how α-Syn proteostasis is maintained at an early age since most patients with familial PD develop pathology and symptoms only after decades.

Mitochondrial dysfunction is another hallmark of PD characterized by loss of mtDNA, increased oxidative stress due to reactive oxygen species (ROS), deterioration of metabolic activities, and reduced calcium homeostasis^9^. Disruption of the electron transport chain complexes with 1-methyl-4-phenyl-1,2,3,6-tetrahydropyridine (MPTP) is sufficient to cause parkinsonian-like symptoms in mice^10^. Furthermore, mutations in genes encoding mitochondrial proteins such as Parkin and PINK1, also cause familial PD^11^. Recent studies suggest that α-Syn interacts with mitochondrial outer membrane import channel proteins TOM20 and TOM40^12,13^. In an α-Syn preformed fibril (PFF)-treated mouse model of PD, the majority of a pathogenic form of α-Syn (serine 129 phosphorylated α-Syn) binds to mitochondria and alters cellular respiration^14^. α-Syn also interacts with the Voltage-Dependent Anion Channel (VDAC) of the mitochondrial outer membrane in HeLa cells^15^. In addition to mitochondrial outer membrane proteins, α-Syn also interacts selectively with cardiolipin in mitochondria^16^. A recent study suggests that the conversion of monomeric α-Syn into toxic oligomeric states preferentially occurs on mitochondrial membranes via interactions with mitochondrial cardiolipin^17^. Moreover, α-Syn has been detected in the mitochondrial intermembrane space^18^ and interacts with complex I, causing reduced complex I activity and increased production of ROS^19,20^. Artificial targeting of α-Syn into mitochondria by linking it with a mitochondrial targeting sequencing impaired mitochondrial respiration in human dopaminergic neurons, arguing for the toxicity of accumulating α-Syn in mitochondria^21^. However, it is unclear whether the import of α-Syn into mitochondria is a spontaneous process or occurs only under pathogenic conditions.

The budding yeast *Saccharomyces cerevisiae* has been a useful model for understanding how α-Syn may perturb basic cellular functions that could be extended to human cells^22–25^. These studies demonstrated that α-Syn interferes with a broad range of membrane-based processes to exert its toxicity, such as lipid metabolism, vesicular trafficking, Ca^2+^ and Mn^2+^ transport, protein quality control, and mitochondrial functions^26,27^. In this study, we examined α-Syn in mitochondria using a split-GFP system (spGFP)^28^, which allows α-Syn to be specifically visualized once it enters the mitochondrial matrix in vivo, in contrast to lengthy biochemical fractionation during which the non-native mitochondrial protein could be degraded. We implemented this system in yeast and cultured mammalian cells, as well as in mice, by tagging the endogenous SNCA gene with a part of the spGFP. We show that α-Syn can be constitutively imported into mitochondria in these diverse models. The mitochondrial pool of α-Syn forms puncta but can also be efficiently degraded by specific mitochondrial proteases. The mitochondria-associated α-Syn interacts with proteins comprising diverse pathways and leads to mitochondrial defects similar to those reported for PD neurons. Enhancing mitochondrial degradation of α-Syn rescues the observed cellular defects and may represent a therapeutic approach to alleviate mitochondrial and cellular toxicity caused by α-Syn.

## Results

### α-Syn is imported into mitochondria in yeast and cultured human cells

**Fig. 1:**
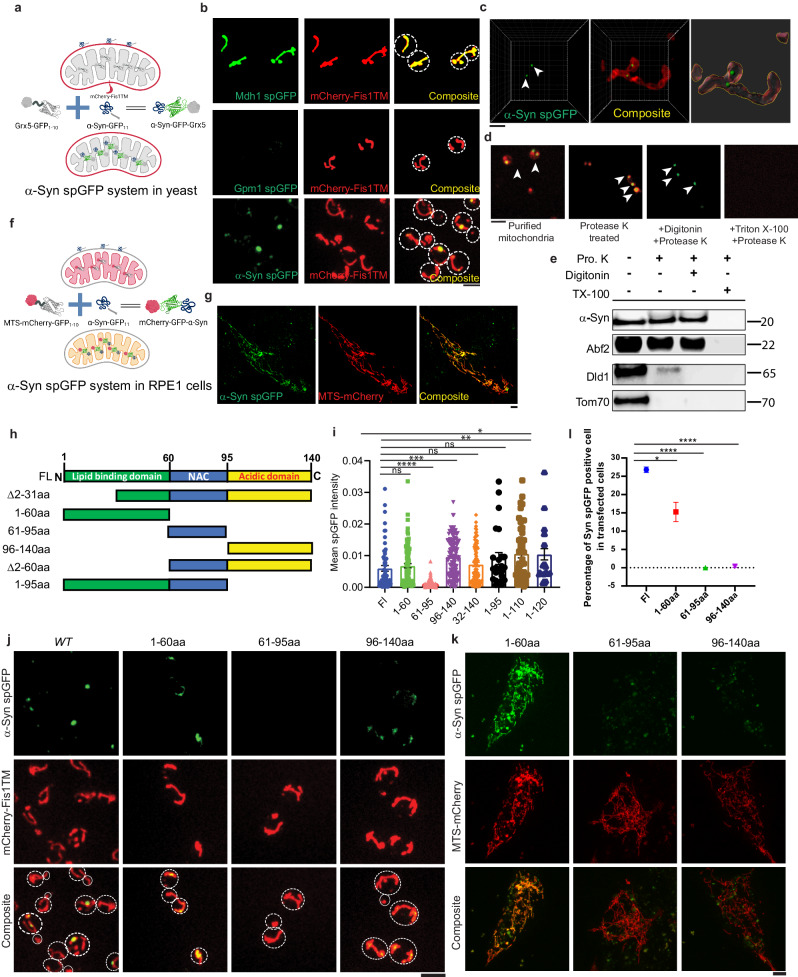
α-Syn is imported into mitochondria in yeast and human cells. **a** Schematic illustration of α-Syn spGFP system in yeast. The mitochondrial outer membrane was labeled with mCherry-tagged Fis1 transmembrane domain; the GFP_1–10_ was targeted to the mitochondrial matrix by tagging endogenous Grx5 and the 11th β-strand of GFP (GFP_11_) was introduced to the COOH terminus of α-Syn. **b** Representative confocal images of log-phase live yeast cells labeled with the mCherry-Fis1TM and Grx5-GFP_1–10_. Endogenous Mdh1 (top), Gpm1 (middle), and a GAP promoter-driven human α-Syn (bottom) were tagged with GFP_11_. Cell outlines were circled with dashed lines. Scale bars, 5 µm. **c** Airyscan super-resolution imaging of α-Syn spGFP cells as described in (**b**). Arrowheads point to α-Syn spGFP puncta inside mitochondria. Scale bars, 1 µm. The right panel shows the 3D-reconstructed image. **d** Representative images of the protease protection assay using mitochondria purified from α-Syn spGFP cells as described in (**b** and **c**). Purified mitochondria were treated with protease K and indicated detergents for 35 min. Arrowheads point to α-Syn spGFP puncta inside mitochondria. Scale bars, 5 µm. **e** Immunoblots of purified mitochondria treated with or without detergents and proteases as indicated. Pro. K, protease K. TX-100, triton X-100. **f** Schematic illustration of α-Syn spGFP system in RPE1 cells. The mitochondrial targeting sequence (ATP synthase Subunit 9, Su9) targeted mCherry linked with GFP_1–10_ (MTS-mCherry-GFP_1–10_) to the mitochondrial matrix and human α-Syn was tagged with GFP_11_ (α-Syn-GFP_11_). **g** Representative confocal images of RPE1 cells transfected with α-Syn spGFP systems. Scale bars, 5 µm. **h** Schematic illustration of three structural domains of α-Syn. **i** Quantification of the mean intensity of spGFP signal in α-Syn truncations spGFP strains. Shown are the Means ± SEM of each cell from three biological repeats. The unpaired two-tailed *t*-test between α-Syn full-length (Fl) and truncations. **j** Confocal live-cell imaging of cells expressing Cherry-Fis1TM, Grx5-GFP_1–10,_ and truncated forms of α-Syn as indicated linked with GFP_11_. Cell outlines were circled with dashed lines. Scale bars, 5 µm. **k** Confocal imaging of live RPE1 cells expressing the α-Syn spGFP system with indicated α-Syn truncations. Scale bars, 5 µm. **l** Quantification of the percentage of α-Syn spGFP positive cells by flow cytometry. Shown are Means ± SEM from three biological repeats. The unpaired two-tailed *t*-test between α-Syn full-length (Fl) and truncations is indicated in the figure.

To visualize the entry of α-Syn into the mitochondrial matrix, we took advantage of the spGFP system^28,29^, in which the eleventh β-strand of GFP (GFP_11_) was introduced to the COOH terminus of human α-Syn (α-Syn-GFP_11_). In yeast, the other part of GFP containing the first ten β-strands (GFP_1–10_) was targeted to the mitochondrial matrix by linking to the COOH terminus of a mitochondrial matrix protein Grx5^29^. The mitochondrial outer membrane was labeled with Fis1 transmembrane domain tagged with mCherry^30^. α-Syn-GFP_11_ was expressed in yeast using the constitutive GAP promoter, which allows cells to grow in the normal glucose-containing medium, and α-Syn imported into the mitochondrial matrix enables reconstitution of GFP fluorescence (Fig. 1a). Confocal live-cell imaging showed that α-Syn spGFP fluorescence colocalized with mitochondria (Fig. 1b). As positive and negative controls in the Grx5-GFP_1–10_ background, the GFP_11_-tagged mitochondrial matrix protein Mdh1 or cytosolic protein Gpm1 showed strong or no GFP signal, respectively, in mitochondria (Fig. 1b). The expression levels of α-Syn and Gpm1 were comparable (Supplementary Fig. 1a). Super-resolution microscopy further showed that α-Syn formed puncta within mitochondria (Fig. 1c), and these puncta colocalized with the mitochondrial matrix disaggregase Hsp78 (Supplementary Fig. 1b), suggesting that α-Syn aggregates in mitochondria. To biochemically confirm the presence of α-Syn in the mitochondrial matrix, we purified mitochondria from the yeast strain above and performed a protease protection assay (Fig. 1d, e). Digitonin and Triton X-100 were used to permeabilize the mitochondrial outer membrane and inner membrane, respectively, and protease K treatment to degrade α-Syn that was not protected by membranes. Immunoblot analysis showed the presence of α-Syn in both the mitochondrial intermembrane space and the mitochondrial matrix (Fig. 1d, e).

We also applied the α-Syn spGFP system to human RPE1 cells by expressing α-Syn tagged with GFP_11_ and mitochondrial matrix-targeted mCherry-GFP_1–10_ (MTS-mCherry-GFP_1–10_)^31^ and found that α-Syn was present in mitochondria by imaging, and this occurred in 26.8% of transfected cells by flow cytometry analysis. (Fig. 1f, g, l).

To determine which domain of α-Syn was essential for accumulation inside mitochondria, each of the three α-Syn structural domains^5,6^ (N-terminal lipid binding, NAC domain, and C-terminal unstructured domain) were tagged with GFP_11_ and co-expressed with Grx5-GFP_1–10_ in yeast (Fig. 1h–j). Both the N-terminal and C-terminal domains on their own, but not the central NAC domain, were imported into mitochondria constitutively (Fig. 1i, j). Fragments containing the N-terminal domain plus the NAC domain, or the NAC domain plus the C-terminal domain, were also imported into mitochondria (Fig. 1i and Supplementary Fig. 1c–e), suggesting that either N-terminal or C-terminal is sufficient to be imported into mitochondria. We further tested if the above mechanisms found in yeast were conserved in humans. The N-terminal domain, but not the NAC or C-terminal domain, was imported into mitochondria in RPE1 cells, although the N-terminal domain was imported less efficiently than full-length α-Syn (Fig. 1k, l). However, because the protein expression level of the C-terminal domain was exceedingly low compared with full-length α-Syn, which could be caused by instability of the C-terminal domain, the lack of spGFP signal in mitochondria may not reflect an import defect (Fig. 1k, l and Supplementary Fig. 1f, g).

### α-Syn accumulates in the mitochondria of dopaminergic neurons and microglia in mouse brains in an age-dependent manner

**Fig. 2:**
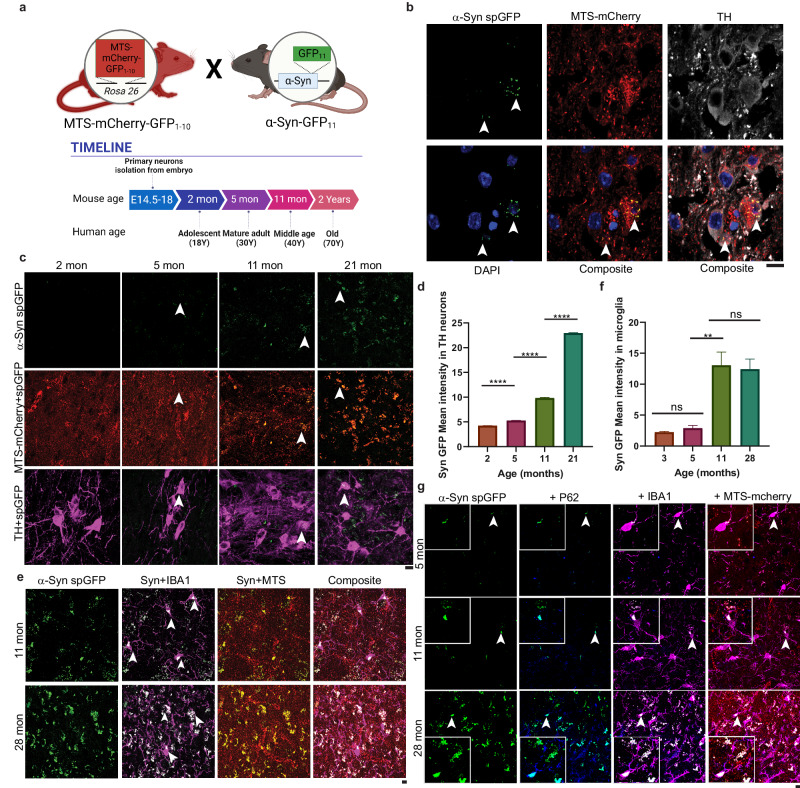
α-Syn import into mitochondria is age-dependent in transgenic α-Syn spGFP mouse. **a** Schematic illustration of α-Syn spGFP transgenic mouse and comparison of relative ages between mice and humans. The 3’ terminus of the endogenous *SNCA* ORF was tagged with GFP_11_ and the mitochondrial matrix-targeted mCherry-GFP_1–10_ (MTS-mCherry-GFP_1–10_) under CAG promoter was inserted into the *Rosa26* locus, both *via* CRISPR-Cas9 genome editing. **b** Airyscan super-resolution confocal images of midbrain sections of 5-month-old α-Syn spGFP transgenic mouse stained with TH marker. Arrowheads point to α-Syn spGFP signal inside mitochondria. **c** Representative confocal images of midbrain sections of 2-, 5-, 11-, and 21-month-old α-Syn spGFP mice. Arrowheads point to α-Syn spGFP inside mitochondria in TH-positive cells. **d** Quantification of the mean intensity of α-Syn spGFP signal in TH-positive cells. ****P < 0.0001; one-way ANOVA followed by Tukey’s multiple comparison test. The unpaired two-tailed *t*-test between each age from three biological repeats. **e** Representative confocal images of brain sections of 11- and 21-month-old α-Syn spGFP transgenic mouse stained with IBA1. Arrowheads point to α-Syn spGFP puncta inside mitochondria in IBA1-positive cells**. f** Quantification of the mean intensity of α-Syn spGFP signal in IBA1-positive microglia. Shown are Means ± SEM of spGFP intensity in arbitary unit from three biological repeats. **P = 0.0029; NS non-significant; one-way ANOVA followed by Tukey’s multiple comparison test. The unpaired two-tailed *t*-test between ages. **g** Representative confocal images of brain sections of 5-, 11-, and 28-month-old α-Syn spGFP transgenic mouse stained with IBA1 and P62. Arrowheads point to zoomed-in regions where α-Syn spGFP puncta-containing mitochondria colocalized with P62 in microglia. Scale bars, 10 µm.

To examine if the endogenous α-Syn is imported into mitochondria in vivo of mammals, we generated a transgenic mouse model in which the COOH terminus of endogenous *SNCA* gene was tagged with GFP_11_, and MTS-mCherry-GFP_1–10_ under CAG promoter was ubiquitously expressed by inserting into the *Rosa26* locus, both *via* CRISPR-Cas9-mediated genome editing (Fig. 2a). This mouse model allows visualization of mitochondrial import of α-Syn in different tissues and cell types during mouse aging (Fig. 2a). MTS-mCherry-GFP_1–10_ fluorescence emitted by mCherry also colocalized with a mitochondrial protein TOM20 by immunofluorescence in mouse brain slices (Supplementary Fig. 2d). Compared to the wild-type, α-Syn spGFP-tagged mouse brain showed a moderate reduction in the protein level of α-Syn (Supplementary Fig. 2f, g). Nonetheless, by confocal imaging of spGFP fluorescence in tissue slices, we detected the accumulation of α-Syn spGFP signal in mitochondria of mouse brain in 5-month-old mice (Fig. 2b). The mitochondria-associated α-Syn spGFP signal was enriched in the midbrain and was present in cells that stained positive for tyrosine hydrolase (Th), a marker specific for dopaminergic neurons (Fig. 2b). Interestingly, the accumulation of α-Syn in mitochondria in Th^+^ neurons correlated with age: whereas the spGFP signal was low in 2- and 5-month-old animals, it increased dramatically in 11- and 21-month-old mouse brains (Fig. 2c, d). Furthermore, in the brains of old animals, α-Syn spGFP was frequently observed in Th^-^ cells (Fig. 2c and Supplementary Fig. 2a, b). Using different cell type markers, we found that the α-Syn accumulated in mitochondria of microglia marked by IBA1 (Ionized calcium binding adapter molecule 1), but not in cells stained with astrocyte maker GFAP (Fig. 2e, f and Supplementary Fig. 2c, e). In microglia, some of the large puncta containing α-Syn spGFP and MTS-mCherry colocalized with p62, an autophagosome marker, in 11 and 28, but not 5-month-old brains (Fig. 2g), suggesting that mitophagy of the α-Syn-containing mitochondria were increased in aged cells.

### α-Syn interacts with functional complexes in mitochondria and causes mitochondrial dysfunction

**Fig. 3:**
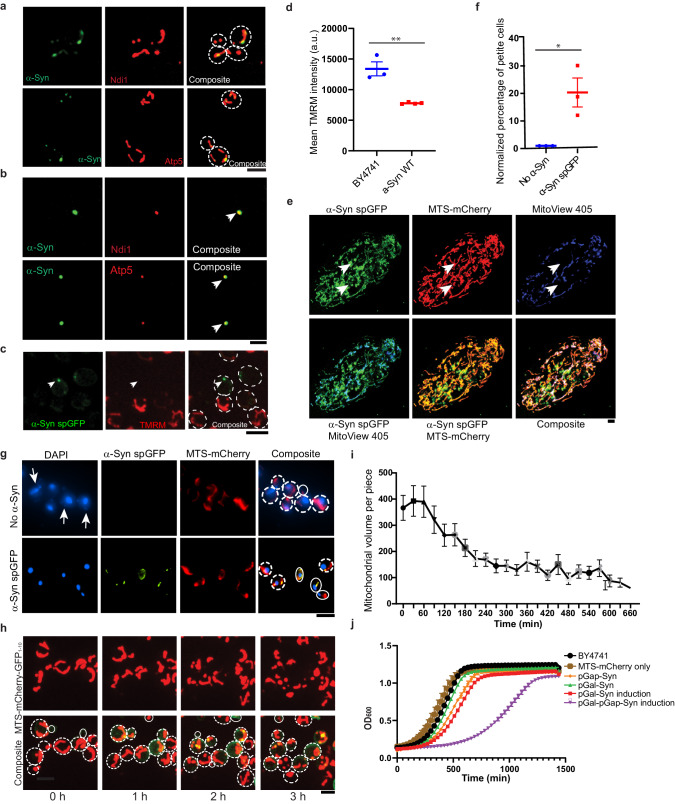
Mitochondrial dysfunctions caused by α-Syn. **a** Confocal images of live yeast cells expressing α-Syn spGFP and mScarlet II-tagged Ndi1 or Atp5. Cell outlines were circled with dashed lines. **b** Confocal images of cell lysates in buffer containing 1% Triton X-100 showing that α-Syn stably associated with Ndi1 and Atp5 in aggregates (arrowheads). **c** Confocal images of mitochondrial membrane potential staining by using TMRM. α-Syn was linked with GFP_11_ and the mitochondrial matrix protein Grx5 was tagged with GFP_1–10_. Arrowhead points to an α-Syn spGFP punctum inside mitochondria without a TMRM signal. Cell outlines were circled with dashed lines. **d** Mitochondrial membrane potential was detected with TMRM and measured by FACS. Each data point represents the mean TMRM intensity from a biological repeat. Shown are Means ± SEM from 3–4 biological repeats with unpaired two-tailed *t*-test. **e** Confocal images of MitoView 405 dye staining in live RPE1 cells expressing the α-Syn spGFP system. Arrowheads point to α-Syn spGFP puncta inside mitochondria with diminished MitoView 405 signal, which is a blue-fluorescent mitochondrial membrane potential dye in live cells. **f** The percentage of respiration-deficient cells (petites) in α-Syn spGFP cells normalized to cells expressing MTS-mCherry only, measured by the TTC staining in yeast. Shown are Means ± SEM from 3 biological repeats with unpaired two-tailed *t*-test. **g** DAPI staining for white or pink colonies picked from TTC staining. DAPI stains both nuclear and mitochondrial DNA. Arrowheads point to mtDNA. Cell outlines were circled with white lines. **h** Time-lapse movie showing that acute induction of α-Syn caused mitochondrial fragmentation in yeast. Total mitochondria were labeled with mCherry-Fis1TM; α-Syn was tagged with GFP_11_, and mitochondrial matrix protein Grx5 was tagged with GFP_1–10_. Cell outlines were circled with white lines. **i** Quantification of total mitochondrial volume per cell change over time of movies in H. Shown are Means ± SEM of mitochondrial volume (*n* = 28). **j** Growth curves of cells expressing α-Syn under GAP promoter (pGAP-α-Syn), Gal promoter with the GEM system (pGal-α-Syn) or both (pGAP-α-Syn; pGal-α-Syn). β-estradiol was used to induce the expression of α-Syn in the GEM system. OD600 was recorded every 30 min at 30 °C. Shown are Means ± SEM from three biological repeats. Scale bars, 5 µm.

α-Syn was reported to impair mitochondrial functions through its interactions with components of the electron transport chain^19^, and its interaction with complex I reduced complex I activity and increased ROS production. To verify the interaction of α-Syn with the oxidative phosphorylation machineries, we expressed α-Syn spGFP system in strains expressed mScarlet-I labeled NDI1, a NADH-ubiquinone oxidoreductase^32^ or ATP5^33^, a component of Complex V, separately (Fig. 3a). After disrupting the outer and inner membranes of purified mitochondria with 1% Triton X-100 containing lysis buffer, Atp5 and Ndi1 remained associated with α-Syn puncta (Fig. 3b). Consistently, the mouse ortholog of ATP5, Atp5o, also colocalized with α-Syn puncta in 11-month-old mouse brains (Supplementary Fig. 3e).

Since the electron transport chain is important for producing the proton gradient across the mitochondrial inner membrane, we measured mitochondrial membrane potential (ΔΨ) with Tetramethylrhodamine (TMRM) staining^34^. Mitochondria with α-Syn accumulation showed a marked decline in ΔΨ (Fig. 3c, d). Similarly, α-Syn overexpression also reduced mitochondrial membrane potential in RPE1 cells by staining with MitoView405, which is for monitoring changes in mitochondrial membrane potential in live cells (Fig. 3e and Supplementary Fig. 3f). We also measured the frequencies of respiration-deficient cells, which indicate the loss of mtDNA using 2,3,5-triphenyltetrazolium chloride (TTC) staining^35^ in yeast expressing α-Syn. DAPI staining confirmed the loss of mtDNA in TTC-negative cells. We found that α-Syn accumulation in the mitochondria of yeast cells resulted in an increased loss of mtDNA (Fig. 3f, g). Furthermore, inducible expression of α-Syn and its subsequent accumulation in mitochondria caused mitochondrial fragmentation in both yeast and RPE1 cells (Figs. 3h, i, 5g and Movie S1). Although α-Syn under the constitutive GAP promoter in yeast cells did not significantly affect cell growth, acute high-level expression from a second copy of α-Syn with a β-estradiol-inducible GEM system^36^ significantly impaired cell growth (Fig. 3j). These data together showed that high-level α-Syn causes mitochondrial dysfunction and cellular fitness decline.

### Imported α-Syn is degraded in mitochondria

**Fig. 4:**
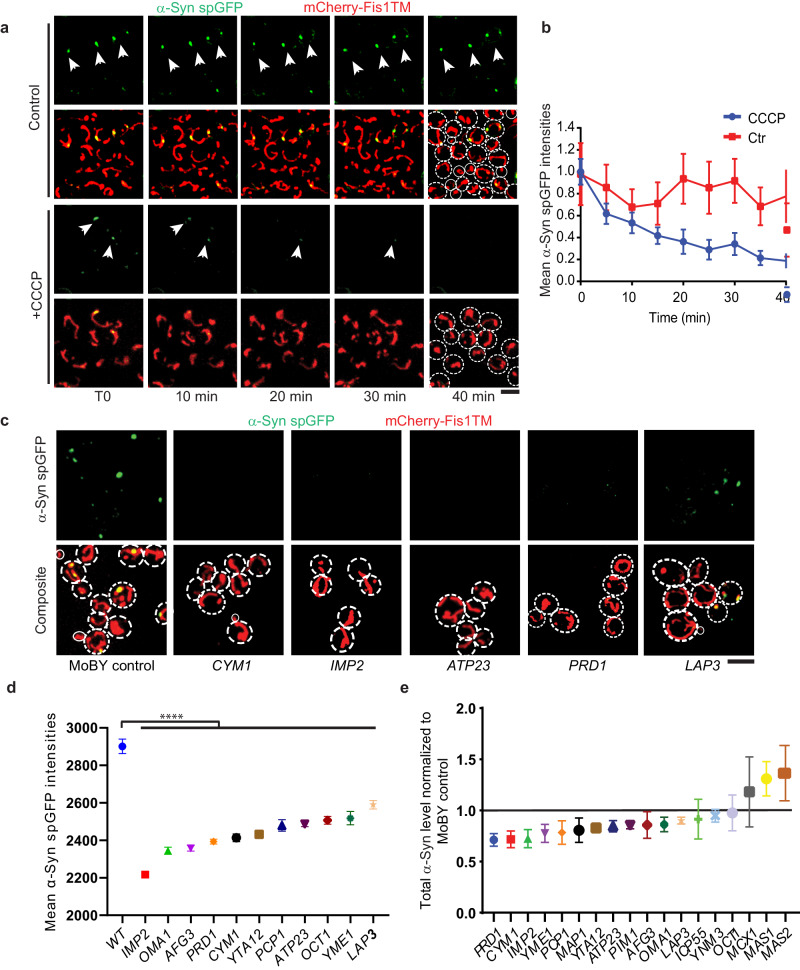
Imported α-Syn is degraded in mitochondria by specific proteases. **a** Time-lapse movie of log-phase α-Syn spGFP system expressing cells with or without CCCP added at the T0 of the movie. The interval time is 5 min. Arrowheads point to α-Syn spGFP puncta inside mitochondria. Scale bars, 5 µm. **b** Quantification of the mean α-Syn spGFP intensity in (**a**). Shown are Means ± SEM of α-Syn spGFP intensity in mitochondria from three biological repeats. ***P < 0.001; two-way ANOVA test. **c** Representative confocal images of cells expressing the α-Syn spGFP system that were introduced with additional copies of genes encoding individual mitochondrial proteases (as indicated in the figure legends) via the MoBY plasmid. Scale bars, 5 µm. **d** Quantification of the α-Syn spGFP intensities in (**c**). Shown are Means ± SEM from 3 biological repeats. ****P < 0.0001; one-way ANOVA followed by Tukey’s multiple comparison test. **e** Quantification of α-Syn levels in cells with indicated MoBY plasmid by immunoblots. Pgk1 was used as the loading control.

We used time-lapse imaging to track the fate of α-Syn spGFP in mitochondria over time. When α-Syn was under constitutive expression, the spGFP signal in mitochondria was maintained at a steady level throughout the course of the observation (Fig. 4a, b and Movie S2). When treating these cells with CCCP, which blocked the import of α-Syn, the α-Syn spGFP signal in mitochondria decreased with a half-life of 6.9 min (Fig. 4a, b and Movie S3), while the spGFP signal of the matrix protein Grx5 did not change, suggesting that α-Syn was rapidly degraded in mitochondria (Supplementary Fig. 3g and Movie S4).

To identify mitochondrial proteases responsible for the degradation of α-Syn, we moderately increased the copy number of each of the known mitochondrial proteases by using centromeric plasmids from the molecular barcoded yeast (MoBY) library, in which each gene is controlled by its native promoter and terminator^37^. Increased copy number of several mitochondrial proteases significantly reduced the steady-state level of α-Syn spGFP, but not Grx5-spGFP, in mitochondria (Fig. 4c–e and Supplementary Fig. 3a, b, d). Plasmids expressing Cym1 and Prd1 had the strongest effect on reducing α-Syn spGFP accumulation in mitochondria, whereas deletion of *CYM1* or *PRD1* significantly increased the accumulation of α-Syn in mitochondria (Fig. 4e and Supplementary Fig. 3c). On the other hand, overexpression of Pim1, Lon protease found to be involved in aggregate dissolution after heat shock^29^, or Nma111/Ynm3, a serine protease whose human ortholog HtrA2 has genetic association with PD^38^, did not affect α-Syn accumulation in mitochondria (Fig. 4e and Supplementary Fig. 3a).

### Enhancing specific mitochondrial proteolytic activities reduces α-Syn toxicity

**Fig. 5:**
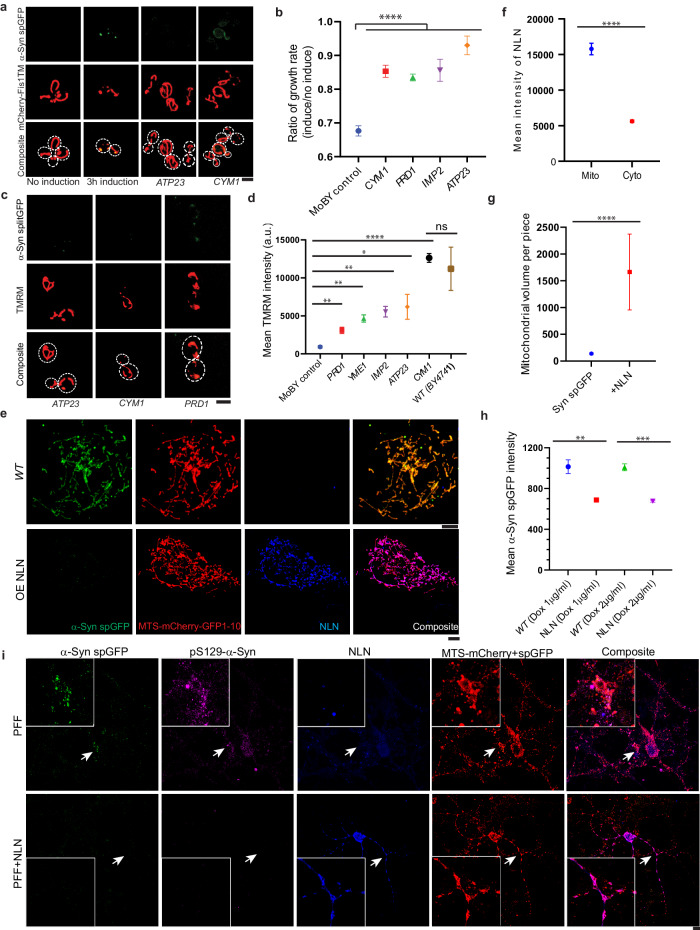
Enhancing α-Syn degradation in mitochondria reduces α-Syn toxicity. **a** Representative confocal images of yeast cells expressing the GEM inducible α-Syn spGFP system that were introduced with additional gene copies of individual mitochondrial proteases via MoBY plasmids. **b** Ratio of growth rates (add estradiol/no estradiol) for cells expressing α-Syn under both the GAP promoter and the estradiol-GEM inducing promoter (pGAP-α-Syn; pGal-α-Syn) with extra gene copies of the indicated protease. Shown are Means ± SEM from 3 biological repeats. The unpaired two-tailed *t*-test between control and MoBY strains. **c** Representative confocal images of mitochondrial membrane potential measured by TMRM staining for cells as in (**b**). **d** Quantification of mean TMRM intensity in strains with extra gene copies of the indicated protease. Shown are Means ± SEM (*n* ≥ 20 cells) with unpaired two-tailed *t*-test. **e** Representative confocal images of RPE1 cells stably expressing α-Syn spGFP with or without NLN overexpression. **f** Quantification of the mean NLN intensity in mitochondria and cytosol in α-Syn spGFP stable cells with overexpressing of NLN. Shown are Means ± SEM (*n* ≥ 20 cells) from 3 biological repeats. ****P < 0.0001; two-way ANOVA test. **g** Quantification of mitochondrial volume in α-Syn spGFP stable cells with or without overexpressing of NLN. Shown are Means ± SEM (*n* ≥ 20 cells) with unpaired two-tailed *t*-test from 3 biological repeats. **h** Quantification of the mean α-Syn spGFP intensity in RPE1 cell lines stably expressing dox-inducible α-Syn-GFP_11_ with or without NLN overexpression. Shown are Means ± SEM with unpaired two-tailed *t*-test from 3 biological repeats (*n* ≥ 20 cells). **i** Representative confocal images of primary neurons from α-Syn spGFP mouse with or without NLN overexpression for 7 days post α-Syn PFF treatment. Arrowheads point to α-Syn spGFP puncta inside mitochondria under PFF treatment (in the first row) or zoomed-in regions with overexpression of NLN (in the second row). Scale bars, 5 µm.

To determine whether elevating the level of mitochondrial proteases that can degrade α-Syn would reduce the cellular toxicity and mitochondrial defects caused by α-Syn expression, we moderately increased the gene copy number using the aforementioned MoBY system. Cym1, Prd1, Imp2 or Atp23 proteases showed the highest inhibitory effects on α-Syn accumulation in mitochondria (Fig. 4c, d) and rescued growth defects caused by the induced high-level expression of α-Syn (Fig. 5a, b). Moreover, increasing copy numbers of these mitochondrial proteases also restored mitochondria membrane potential in α-Syn-expressing cells (Fig. 5c, d).

To examine if increasing the level of Prd1 homolog could rescue mitochondrial defects in human cells, we generated RPE1 cell lines expressing dox-inducible α-Syn spGFP system with or without stable ectopic expression of NLN (Prd1 ortholog) under the PFFV promoter (Supplementary Fig. 3h, i). The overexpressed NLN is predominately localized to mitochondria in RPE1 cells, as confirmed by immunostaining (Fig. 5e, f), significantly reduced α-Syn accumulation in mitochondria and mitochondrial fragmentation caused by the induced high-level expression of α-Syn (Fig. 5e, g, h). To further test whether overexpression of NLN could rescue PD pathologies in a neuronal model of synucleinopathy, we applied preformed fibrils of mouse α-Syn (α-Syn PFF)^39^ to primary neurons from the α-Syn spGFP transgenic mouse. PFF seeds the recruitment of endogenous α-Syn into pSer129-α-Syn-positive aggregates that recapitulate features of the Lewy bodies and Lewy neurites in PD^40^. Primary cortical neurons from α-Syn spGFP mouse were isolated and treated with α-Syn PFF for 14 days starting at 7 days in vitro (DIV7). Compared to the control, overexpression of NLN decreased α-Syn accumulation in mitochondria as well as pSer129-α-Syn aggregates in PFF-treated primary neurons (Fig. 5i and Supplementary Fig. 3j). Collectively, our results suggest that enhancing the level of NLN rescues defects caused by synucleinopathy in yeast, human cells, and mouse primary neurons.

## Discussion

The data presented above have shed light on a direct link between α-synucleinopathies and mitochondrial dysfunction, two hallmarks of PD. We show that the α-Syn import into mitochondria is conserved in yeast, mouse, and human cells. During mouse aging, α-Syn accumulation in mitochondria of DA neurons and microglia gradually increased, which helps explain why age is a risk factor for PD^41^. The decline of proteostasis during aging may lead to increased import of misfolded proteins into mitochondria, including α-Syn. This could overwhelm the proteolytic apparatus, leading to the accumulation of misfolded proteins in mitochondria, which further compromises mitochondrial functions^21^. Dysfunctional mitochondria would have reduced contribution to maintaining cytosolic proteostasis and cause metabolic stress, leading to further accumulation of α-Syn and other misfolded proteins in the cytosol, forming protein aggregates such as Lewy bodies. This vicious cycle may lead to neuronal death.

A recent study targeted α-Syn directly into mitochondria with MTS, which severely damaged mitochondrial functions in DA neurons^21^, confirming the detrimental effects of accumulating α-Syn in mitochondria. We note that the spGFP reporter only detects the mitochondrial pool of α-Syn and cannot assess the relative portion of α-Syn in mitochondria comparing to cytosol, however, our data show that even small amount of accumulation of α-Syn in mitochondria could significantly damage mitochondrial functions. In *C. elegans*, neurons can dispose mitochondria and protein aggregates in exophers^42^. It will be interesting to determine if there is a similar mechanism in mammals that would help remove α-Syn-containing mitochondria in a non-cell-autonomous fashion, when mitophagy failed to clear them in neurons. Recent studies found that microglia can uptake α-Syn released from neurons^43^, and mitochondria can be transferred between microglia^44^. It is currently unclear if the α-Syn-containing mitochondria accumulated in microglia during aging originate from neurons or arise directly in microglia cells caused by high levels of α-Syn accumulation in mitochondria.

Our data also suggest that enhancing certain mitochondrial proteases rescues both α-Syn toxicity and mitochondrial dysfunction. Other studies have shown that enhancing mitochondrial proteostasis reduces Aβ proteotoxicity^45,46^ and extends the lifespan of worms^47^. These findings suggest that rescuing mitochondrial proteostasis can antagonize age-associated defects. On the contrary, inhibiting mitochondrial proteases, such as HtrA2 and Lon protease, significantly aggravates α-Syn seeding, as well as amyloid-β 1–42 (Aβ42) aggregation in human neuroblastoma cells (SH-SY5Y)^48^. Our data suggest that increasing the levels of specific mitochondrial proteases such as Neurolysin significantly reduced the accumulation of α-Syn in mitochondria and rescued the mitochondrial dysfunction brought about by α-Syn overexpression. The variable expression of different mitochondrial proteases that are responsible for the degradation of imported α-Syn may help explain the cell-type-specific vulnerability of α-synucleinopathies and mitochondrial dysfunction in PD. By analyzing the GTEx RNA-seq database^49^ and a published single-cell RNA-seq dataset^50^, we found that the transcript level of Neurolysin is increased in substantia nigra where DA neurons are enriched during normal human aging (Supplementary Fig. 4a), but reduced in DA neurons derived from induced pluripotent stem cell (iPSC) carrying the GBA-N370S PD risk variant compared to the healthy control^50^ (Supplementary Fig. 4b). Therefore, the reduced expression of Neurolysin in DA neurons may explain the DA neuron-specific toxicity of α-synucleinopathies and mitochondrial dysfunction in PD. Further study is required to determine if increasing Neurolysin levels or stimulating its proteolytic activity in DA neurons of aged brains could reduce α-Syn accumulation in mitochondria and hereby alleviate mitochondrial dysfunction and neuronal death in PD.

## Methods

### Yeast strains, plasmids, and culture media

Yeast strains of the BY4741 background (MATa his3Δ1 leu2Δ0 met15Δ0 ura3Δ0) were used in this study, and the detailed strain genotypes are listed in Supplementary Table 1. Gene deletion and tagging (HA, mcherry, GFP_11_, and GFP) in this study were performed with PCR-mediated homologous recombination^51^ or picked from the Yeast KO Collection^52^, and verified by PCR genotyping. Both GFP_11_ and MTS–mCherry–GFP_1–10_ plasmids of yeast and mammalian systems were from our previous study^29^. GFP_1–10_ was in frame with the mitochondria targeting sequence (MTS) and mCherry in MTS-mCherry-GFP_1–10_ constructs or used for tagging of the endogenous Grx5. The MoBY library^37^ was purchased from Dharmacon Reagents (Catalog ID: YSC5432). The mSC-II library was a gift from Michael Knop lab^33^. Cells were grown in YPD for biochemistry and growth assays, in synthetic complete (SC) medium for imaging. The respective media contained either 2% glucose (YPD, SC-complete) or 2% galactose (YPGal, SC-Glucose + Gal). OD_600_ was used to estimate the amount of yeast cells used in various experiments.

### Animals

Parental mice C57BL/6J for making the α-Syn spGFP transgenic mice were obtained from the Jackson Laboratories (strain #000664). All mice housing, breeding, and experiment procedures were performed according to the guidelines of the Laboratory Animal Manual of the National Institute of Health Guide to the Care and use of Experimental Animals and were approved by Johns Hopkins University Animal Care and Use Committee. Mice were housed in a 12 h light/dark cycle with free access to food and water. Randomized mixed-gender groups were used for animal experiments.

The α-Syn spGFP transgenic mice were generated and characterized in this study, using homology-directed repair-based, CRISPR-Cas9-induced precise gene editing as described below. CAG promoter-driven MTS (Su9)-mCherry-GFP_1–10_ sequence was inserted into the safe harbor *Rosa26* locus using a plasmid containing the construct flanked by *ROSA26* homologous sequence (1083 bp upstream/4341 bp downstream overlap). They were microinjected together with CRISPR Cas9 protein and crRNA (CGCCCATCTTCTAGAAAGAC) into one-cell embryos of C57BL/6J mice (strain #000664 from the Jackson Laboratory) by the Transgenic Core Laboratory in Johns Hopkins University, resulting in the MTS-mCherry-GFP_1–10_ mice. PCR genotyping was performed with primer pairs forward TTCCCTCGTGATCTGCAACTC and reverse CTTTAAGCCTGCCCAGAAGACT for *WT Rosa*26; forward GTGGGAGCGGGTAATGAACTTT and reverse TCCTGCAATGATGAATCTTGAGTGA for MTS-mCherry-GFP_1–10_ knock-in. The correct insertion generated a band size of 69 bp. GFP_11_ targeted COOH terminus of the endogenous *SNCA* gene was created by using crRNA (CCGGCAGATCTTAGGAGATT), Cas9, and DNA template (GAAGGCTACCAAGACTATGAGCCTGAAGCCGGGGGATCCGGTCGACCCGGAGGCGGTTCTAGAGATCATATGGTTTTGCATGAATATGTTAATGCTGCTGGTATTACT), resulting the α-Syn-GFP_11_ mice. PCR genotyping was performed with primer pairs forward CCTGATATTAGGAAGGCTACCAAGACT and reverse CGCCTCCGGGTCGA for GFP_11_ knock-in; forward CCTGATATTAGGAAGGCTACCAAGACT and reverse GCACTTGTACGCCATGGAAGA for *WT SNCA*. Injections were performed by the Transgenic Core Laboratory at Johns Hopkins University. Crossing α-Syn-GFP_11_ and MTS-mCherry-GFP_1–10_ mice resulted in the α-Syn spGFP mice. Homozygous α-Syn spGFP mice were PCR genotyped and used in this study.

### Antibodies

HA-tag (C29F4): mAb #3724, Cell Signaling Technology. Anti-Tom70 antibody, anti-Dld1 antibody, and anti-Abf2 antibody were kindly provided by S. Claypool’s laboratory (Johns Hopkins University). Anti-mouse IgG, HRP-linked Antibody, Cell Signaling Technology, Cat# 7076. mCherry antibody: PA5–34974, Invitrogen. GAPDH (D16H11) XP® Rabbit antibody: CS#5174S, Cell Signaling. PGK1 Monoclonal Antibody (22C5D8): #459250, Invitrogen. Ms mAb to NLN, ab119802, Abcam. Map2 Alexa Fluor 647 conjugated anti-MAP2, #801806, BioLegend. Iba1/AIF-1(E4O4W) XP Rabbit mAb, #17198 S, Cell Signaling Technology. Rabbit anti-Tyrosine Hydroxylase pAb, NB300–109, NOVUS. Rb mAb to Alpha-synuclein phosphor S129, ab51253, abcam. Purified Mouse Anti-alpha-Synuclein, #610787, BD Biosciences. Purified anti-GFAP, #801103, BioLegend. Purified anti-p62(SQSTM1), #814802, BioLegend. Goat pAb to Ms IgG (Alexa Fluor 405), ab175660, Abcam. Goat pAb to Rb IgG (Alexa Fluor 647), ab150079, Abcam. Goat pAb to Rb IgG (Alexa Fluor 405), ab175652, Abcam. ATP5O Polyclonal antibody, 10994–1-AP, Proteintech. GFP11-tag antibody, #48545, Signalway Antibody. β-Actin (D6A8) Rabbit antibody, #8457 S, Cell Signaling.

### Confocal microscopy and quantification

Live-cell images were acquired using a Yokagawa CSU-10 spinning disc on the side port of a Carl Zeiss 200 M inverted microscope. Laser 488/561 nm excitation was applied to excite GFP/mCherry, respectively, and the emission was collected through the appropriate filters onto a Hamamatsu C9100–13 EMCCD on the spinning disc confocal system. For multi-track acquisition, the configuration of alternating excitation was applied to avoid the bleed-through of GFP. The spinning disc was equipped with a 100×1.45 NA Plan-Apochromat objective and a 63×1.4 oil Plan-Apochromat objective, respectively. For 3D imaging, 0.5-μm step size for 5–6 μm in total in Z for yeast cells; 1-μm step size was applied for human cells. Images were acquired using MetaMorph (version 7.0; MDS Analytical Technologies) on the CSU-10 spinning disc system. Zeiss LSM880-Airyscan FAST Super-Resolution microscopy equipped with 63×/1.4 PlanApo oil was used for yeast, human cells, and mouse brain sections. The super-resolution images were generated by Airyscan processing.

Live yeast cells imaging: yeast cells were cultured in SC medium overnight at 30 °C. Then they were refreshed in SC medium for at least 3 h at 30 °C to an OD_600_ around 0.1–0.25. For 3D time-lapse imaging, cells were laid on a SC-complete agarose gel pad or cultured in a MatTek (P35G-0–14-C) glass bottom dish that were treated with Concanavalin A (MP Biomedicals, Cat# IC150710.2). Each z series was acquired with a 0.5-μm step size. For GEM inducible systems, 1 M of β-estradiol (E2758–1G, Sigma) was added to the SC-complete medium upon induction. The image processing was performed using the ImageJ software (NIH) or the Imaris software. For visualization purposes, some of the images in the figures were scaled with bilinear interpolation and shown as a max projection on Z for fluorescent channels.

Quantification of spGFP fluorescence was done using a custom Python code that was published in our previous study^29^. In brief, after reading the mCherry and GFP channel *z* stacks, the intensities were summed along the *z*-axis. The resulting 2D image in the GFP channel was then subject to random walk segmentation to segment out the yeast cells from the background and watershed segmentation to separate adjacent cells. The segmentation algorithms were taken from the Scikit image library. After segmentation, the median GFP and mCherry intensities in each cell were calculated. For each cell, the mCherry channel was thresholded at 5% of maximal value in order to detect mitochondria, and median GFP intensity within mitochondria was calculated. This median GFP intensity and mCherry intensity were used in the following analyses. Characterization of mitochondrial volume per piece was derived from the voxel volume of segmented mitochondria.

### Mitochondrial isolation and protease protection assay

Mitochondrial purification was based on a published protocol^53^. In brief, yeast cells expressing α-Syn-HA spGFP were cultured in YPD to an OD_600_ of about 0.3–0.4. Cells were collected by centrifugation and treated with Tris-DTT buffer (0.1 M Tris, 10 mM DTT, adjusted pH to 9.4). After washing with SP buffer (1.2 M sorbital, 20 mM KPi, pH 7.4), cells were treated with 0.5 mg/ml zymolase100T (US Biological) at 30 °C for 40 min. Spheroplasts were then washed with SEH buffer (0.6 M sorbital, 20 mM HEPES-KOH pH 7.4, 2 mM MgCl_2,_ 1 mM EGTA pH 8.0, protease cocktail (P2714, Sigma), 10 µM benzamidine-HCl (B6506), 1 µg/ml 1,10-phenanthroline (P9375), PMSF 1 mM was added before use) and broken with a Dounce homogenizer. The homogenate was centrifuged at 1500×*g* (low speed) for 5 min at 4 °C. Supernatant was collected and centrifuged at 12,000×*g* (high speed) for 10 min at 4 °C. The pellet containing mitochondria was used for protease protection assays.

Purified mitochondria were washed three times with import buffer without ATP (3% w/v fatty acid-free BSA, 250 mM sucrose, 80 mM KCl, 5 mM MgCl_2_, 2 mM KH_2_PO_4_, 10 mM MOPS-KOH, pH 7.2) to remove the protease inhibitor. Then the mitochondria were spun down at 4 °C, 13,000 rpm for 10 min. Mitochondria were resuspended, and the same amount was added to four different 1.5-ml tubes. Group 1 was used as untreated total mitochondria. Group 2 was treated with protease K for 35 min at room temperature to assess protection by the mitochondrial outer membrane. Group 3 was treated with digitonin to permeabilize the outer membrane and Group 4 was treated with Triton X-100 to permeabilize the inner membrane. Then, Groups 3 and 4 underwent the same protease treatment as Group 2. The volume difference was equalized with the SEH buffer. Immediately after the treatment, all the samples were either taken for imaging or treated with PMSF and boiled for 15 min in SDS sample buffer for immunoblotting analysis.

### Mammalian cell culture, transfection, and imaging

Human RPE1 (ATCC CRL4000, authenticated by ATCC based on Ep-16 antigen as determined by flow cytometry using the Ep-16 monoclonal antibody and cytokeratins as determined by immunocytochemistry using a pan-cytokeratin antibody) cells were cultured in Dulbecco’s Modified Eagle Medium: Nutrient Mixture F-12 (DMEM/F12) (GIBCO), supplemented with 10% (v/v) fetal bovine serum (FBS), 100 IU/ml penicillin and maintained at 37 °C with 5% CO_2_ in a humidified incubator. Transient transfections were performed with Lipofectamine 3000 (Invitrogen) according to the manufacturer’s instructions. RPE1 cells were double transfected with MTS–mCherry–GFP_1–10_ and α-Syn tagged with GFP_11_. After 24 or 48 h of transfection, samples were collected for flow cytometry analysis or confocal imaging.

### Lenti vector construction, production, and transduction

The α-Syn spGFP expression lentiviral vectors were generated by cloning the α-Syn-GFP_11_ into a pTRE-Bsd vector and MTS-mCherry-GFP_1–10_ into a pEF1a-Neo vector, respectively. The pSFFV-NLN-Puro plasmid was synthesized by Twist Bioscience. The lentiviral vectors were transfected into HEK293FT cells together with packaging vectors psPAX2 and pMD2.G (1:1.5:1.5) to generate the lentiviruses. The viral supernatants were collected at 48 and 72 h after transfection and concentrated by ultracentrifugation for 2 h at 50,000×*g*. Viral particles were resuspended into a serum-free medium and stored at −80 °C. RPE1 cells were infected with inducible α-Syn spGFP expression lentiviral vectors with or without NLN overexpression, and mouse primary neurons were infected by lentivirus carrying NLN at DIV 6.

### Mouse primary neuronal culture and α-Syn PFF transduction

Primary cortical and midbrain neurons were prepared from E18 pups of α-Syn spGFP transgenic mice and cultured in Neurobasal media supplemented with B-27, 0.5 mM L-glutamine, penicillin and streptomycin (Invitrogen, Grand Island, NY, USA) on cell culture plates or coverslips coated with poly-l-lysine (50 μg/ml, Sigma, #P1024–100MG). The neurons were maintained by changing the medium every 3–4 days. Mouse α-Syn PFF (final concentration 5 µg/mL) and phosphate-buffered saline (PBS) control were added at 7 days in vitro (DIV) and incubated for 10–21 days followed by imaging or biochemical experiments for toxicity assays. Overexpression of NLN by lentivirus transduction was performed one day prior to α-Syn PFF and PBS treatment.

### Immunofluorescence analysis

RPE1 cells were cultured on fibronectin (5 μg/cm^2^, Sigma F2006)–coated glass bottom dishes (MatTek). Mouse primary neurons were cultured on poly-l-lysine (50 μg/ml) coated glass bottom dishes or coverslips. Fixation was performed using 4% paraformaldehyde (PFA) at 30 °C for 15 min, followed by three times PBS washing. Cells were blocked with goat serum 10% in blocking buffer (1× PBS buffer, 0.3% Triton X-100, and 10% goat serum) at room temperature for 1 h. Primary antibody incubation was performed at 4 °C overnight. After washing with three times PBS, cells were incubated with fluorescent secondary antibodies for 1 h at room temperature in the dark. After three times washing with PBS, cells were mounted with a Prolong glass antifade mountant (P36980, Invitrogen) for imaging.

### Immunohistochemistry and quantitative analysis

Mice were transcardiac perfused with ice-cold PBS followed by fixation with 4% paraformaldehyde/PBS (pH 7.4) as described previously with somemodifications^54,55^. Brains were collected and postfixed for 24 h in 4% paraformaldehyde and cryoprotected in 30% sucrose/PBS (pH 7.4) solution for 1–2 weeks in 4 °C. All samples were frozen in an OCT buffer and 20 mm serial coronal sections were cut with a microtome (MICROM HM550). The cryosections were mounted on Superfrost Plus slides. For immunohistochemistry, the cryosections were surrounded by an ImmEdge pen circle and blocked with 10% goat serum/PBS plus 0.3% Triton X-100. Then incubated with primary antibodies, followed by incubation with goat anti-rabbit lgG Alexa fluor 647/405 antibody or goat anti-mouse lgG Alexa fluor 405 antibodies (Abcam). Sections were mounted with Prolong glass antifade mountant (P36980, Invitrogen) and cover slide sealed with nail polish for imaging. The images were taken by Zeiss LSM880 microscope and analysed with ImageJ and Imaris software.

### Tissue lysate preparation

Mice were perfused with ice-cold 1× PBS, the brains were dissected from the skulls. The midbrain tissue was homogenized in a 1× RIPA Buffer (Sigma) and protease/phosphatase inhibitor cocktail (Cell Signaling Technology, 5872S) and lysed with a Dounce homogenizer. Lysates were centrifuged at 14,000 rpm (Eppendorf 5430R) at 4 °C for 30 min and the supernatant was collected. The protein concentration was measured via Pierce BCA protein assay Kit (Thermo 23225) and analyzed by immunoblot.

### Cell lysate preparation

For RPE1 cells: Cells were washed with PBS and scraped and collected for centrifuge at 2000 rpm for 5 min at RT. The cell pellet was resuspended in lysis buffer which includes PIPA buffer supplemented with 0.1% SDS and Protease-Phosphatase inhibitor cocktail. Then the lysate was incubated on ice for 15 min, followed by sonication using QSONICA digital sonicator set as 70 amplitudes, pulse on/off 30 s each cycle for in total 5 min at 4 °C. Then the lysate was incubated on ice for an additional 15 min. The lysate was centrifuged at 13,000×*g* for 5 min at 4 °C and the supernatant was collected. Protein levels were quantified using the Pierce BCA protein assay Kit (Thermo 23225) with BSA standards and analyzed by immunoblot.

For mouse primary neurons: Cells were washed with PBS to remove debris. Cells were scraped in PBS and collected for centrifuge at 2000 rpm for 5 min at RT. Resuspend the cell pellet in lysis buffer (1% Triton X-100 and Protease/Phosphatase inhibitor in PBS) and vortex briefly. Then freeze-thaw on chilled water and dry ice three times with 15 s vortex after each thaw. The samples were centrifuged at 14,000 rpm for 30 min at 4 °C. The supernatants were collected for Triton X-100-soluble fraction. The cell pellet was resuspended in SDS cell lysis buffer (2%SDS, 1% Triton X-100, and Protease-Phosphatase inhibitor in PBS) and pipette several times to dissolve the pellet. Then freeze-thaw on chilled water and dry ice three times with 15 s vortex after each thaw. The samples were centrifuged at 14,000 rpm for 30 min at 4 °C. The supernatants were collected for SDS-soluble fraction. Protein levels were quantified using the Pierce BCA protein assay Kit (Thermo 23225) with BSA standards and analyzed by immunoblot.

For yeast cells: cells were centrifuged at 21,000×*g* for 2 min at 4 °C and removed the supernatants. Cells were washed with 1 ml ddH_2_O and centrifuged again at 21,000×*g* for 2 min at 4 °C. Then removed the supernatant and add 100 µl 1x LDS sample buffer with 40 mM DTT to resuspend cells. The cells were incubated at 100 °C for 10 min and vortexed with 100 µl glass beads for 1 min, then another 5 min incubation at 100 °C. The cells were centrifuged at 21,000×*g* for 2 min at room temperature and collected the supernatant for immunoblot.

### Immunoblot analysis

Electrophoresis on 4–12% or 4–20% gradient SDS-PAGE gel was performed with proteins from mouse brain tissues, primary neurons, RPE1 cells, and yeast cells, as described above. The proteins were then transferred to PVDF membranes using iBlot2 (Thermo Fisher Scientific). The membranes were blocked with blocking solution (Odyssey blocking TBS buffer was used for fluorescent-dye conjugated secondary antibodies and Tris-buffered saline with 5% BSA and 0.1% Tween-20 was used for HRP-conjugated secondary antibodies) for 1 h and incubated with primary antibody at 4 °C overnight. The bands were visualized using Clarity Western ECL substrate (Bio-Rad) or fluorescent-dye conjugated secondary antibodies. Data were acquired using LI-COR imaging systems (LI-COR Biosciences) and analyzed with Image Studio (LI-COR Biosciences). All blots were processed in parallel and derived from the same experiments. Uncropped membranes are available as supplementary information.

### Drug treatment

Cycloheximide (C4859, Sigma) was added to a final concentration of 100 μg/ml. CCCP (C2759, Sigma) was dissolved in DMSO or ethanol to 20 mM as stock and 25 μM was used to treat cells. MG132 (c2211, Sigma) was dissolved in DMSO and 80 μM was used to treat yeast cells.

### Membrane potential measurements

Tetramethylrhodamine methyl ester (TMRM) (Sigma-Aldrich, Cat# T5428–25MG) staining was performed for cells without mCherry labels. Cells were incubated with 2.5 µM TMRM at 30 °C for 10 min and washed three times before recording. Mammalian cells were incubated with 100 nM MitoView™ 405 dye (Biotium, Cat# 70070-T) at 37 °C for 15 min and directly for imaging without washing.

### TTC staining assay

TTC staining assays were performed using the agar medium overlay method^35^. Yeast strains were growing on normal selected plates and cultured at 30 °C for 48 h, then the melted soft agar (1.5%) containing 1 mg/ml TTC was gently poured onto the colonies and incubated at room temperature for 2 h. The TTC staining activity was judged by the color of the colonies, as the white compound can be enzymatically changed to red in normal respiratory-competent cells but not in respiration-deficient cells. The plates were documented by a document scanner and analyzed by ImageJ software.

### Yeast cell fixation and DAPI staining

Yeast cells were cultured in SC medium overnight at 30 °C. Then they were refreshed in SC medium for at least 3 h at 30 °C to an OD_600_ around 0.1–0.25. The cells were spun down and removed medium, added 4% paraformaldehyde, and incubated at room temperature for 15 min. Then washed once with KPO_4_/sorbitol and resuspended in KPO_4_/sorbitol containing 0.2 µg/ml DAPI until imaging by a Nikon Ti-E inverted fluorescence microscope.

### Detergent resistance assay

Log-phase yeast cells cultured in YPD were collected by centrifugation (5000×*g*, 6 min) and then washed once with ddH_2_O, followed by 10 mM DTT treatment for 5 min (pH 9.3) at 30 °C. The cells were then washed with sorbitol buffer (pH 7.5, 1.2 M sorbitol) followed by 8 min digestion with 1 mg/ml Zymolyase 100 T in Zymolyase buffer. The cells then were washed twice with SEH buffer (0.6 M sorbitol, 20 mM HEPES-KOH pH 7.4, 2 mM MgCl_2_, protease cocktail (sigma), PMSF 1 mM was added before use) to remove the Zymolyase and were lysed with a dounce homogenizer. The homogenate was centrifuged at 1500×*g* for 5 min at 4 °C, and then the supernatant was collected and centrifuged at 12,000×*g* for 10 min at 4 °C. The pellet was resuspended and imaged to ensure mitochondria were intact. Then 1% Triton X-100 was added and incubated on ice for 10 min before confocal imaging. The mitochondrial membranes were removed by detergent extraction, which was confirmed by imaging.

### Yeast growth assays

For recording yeast growth, cells of the indicated genotypes were cultured in corresponding media. Overnight cultures were refreshed for 4 h at 30 °C and the OD_600_ of the cells was measured and adjusted to 0.04 in 200 μL and added to a 96-well plate. The wells along the plate’s perimeter were filled with 200 μL medium without sample to avoid evaporation and the plate was sealed with parafilm. The optical density (OD) at 600 nm was continuously monitored at 30 °C using a Tecan Infinite M200 Pro every 30 min. The data were extracted and analyzed using Magellan 7 software and the R package GroFit (https://cran.r-project.org/src/contrib/Archive/grofit/)^56^.

### Flow cytometry

Cells (yeast or mammalian) were analyzed on the Attune NxT flow cytometer (Thermo Fisher Scientific) equipped with appropriate filter sets. Appropriate gating was applied for single-cell populations. Gating for fluorescent reports was based on negative controls. Statistical analysis was performed with GraphPad Prism software.

### RNA-seq analysis

Single-cell RNA-seq data from iPSC-derived DA neurons were obtained from EMBL-EBI Biostudies (Accession # E-MTAB-7303). Raw counts were log normalized with top variable genes according to published protocols^57^. To remove potential technical confounders, we applied f-scLVM and regressed out hidden factors^58^. Beta-Poisson model was used to estimate differential expression between PD and normal cells^59^. Bulk RNA-seq from GTEx V8 brain analysis tissues were obtained from the GTEx portal (https://gtexportal.org/home/datasets). After log normalization, we regressed out covariates similar to the GTEx consortium pipeline including PEER factors, genotype principal components, donor sex, and sequencing batch to obtain normalized expression levels.

### Quantification and statistical analysis

Please refer to the Figure legends or the Method details for a description of the sample size and statistical details. Statistical analysis was performed with Prism 5 (GraphPad Software, La Jolla, USA) and *p* values <0.05 were considered significant.

### Supplementary information

Supplementary Information

Supplementary Fig. 4a

All original data

Supplementary movie 1

Supplementary movie 2

Supplementary movie 3

Supplementary movie 4

## Data Availability

All data needed to evaluate the conclusions are available in the main text or the supplementary materials. Additional data and materials related to this study can be requested from the authors. Requests for resources and reagents should be directed to and will be fulfilled by the corresponding author, RL (rong@jhu.edu). Single-cell RNA-seq data were obtained from EBI (accession E-MTAB-7303). Bulk RNA-seq data was obtained from GTEx v8 (https://gtexportal.org/home/datasets). No new datasets were generated.
