# Supplementary material for: Enhancing mitochondrial proteolysis alleviates alpha-synuclein-mediated cellular toxicity
Source: NPJ Parkinsons Dis. 2024 Jun 21;10:120. doi: 10.1038/s41531-024-00733-y (PMC11192938; doi:10.1038/s41531-024-00733-y)
Supplement: Supplementary file 1 — Supplementary Information [file 41531_2024_733_MOESM1_ESM.pdf]

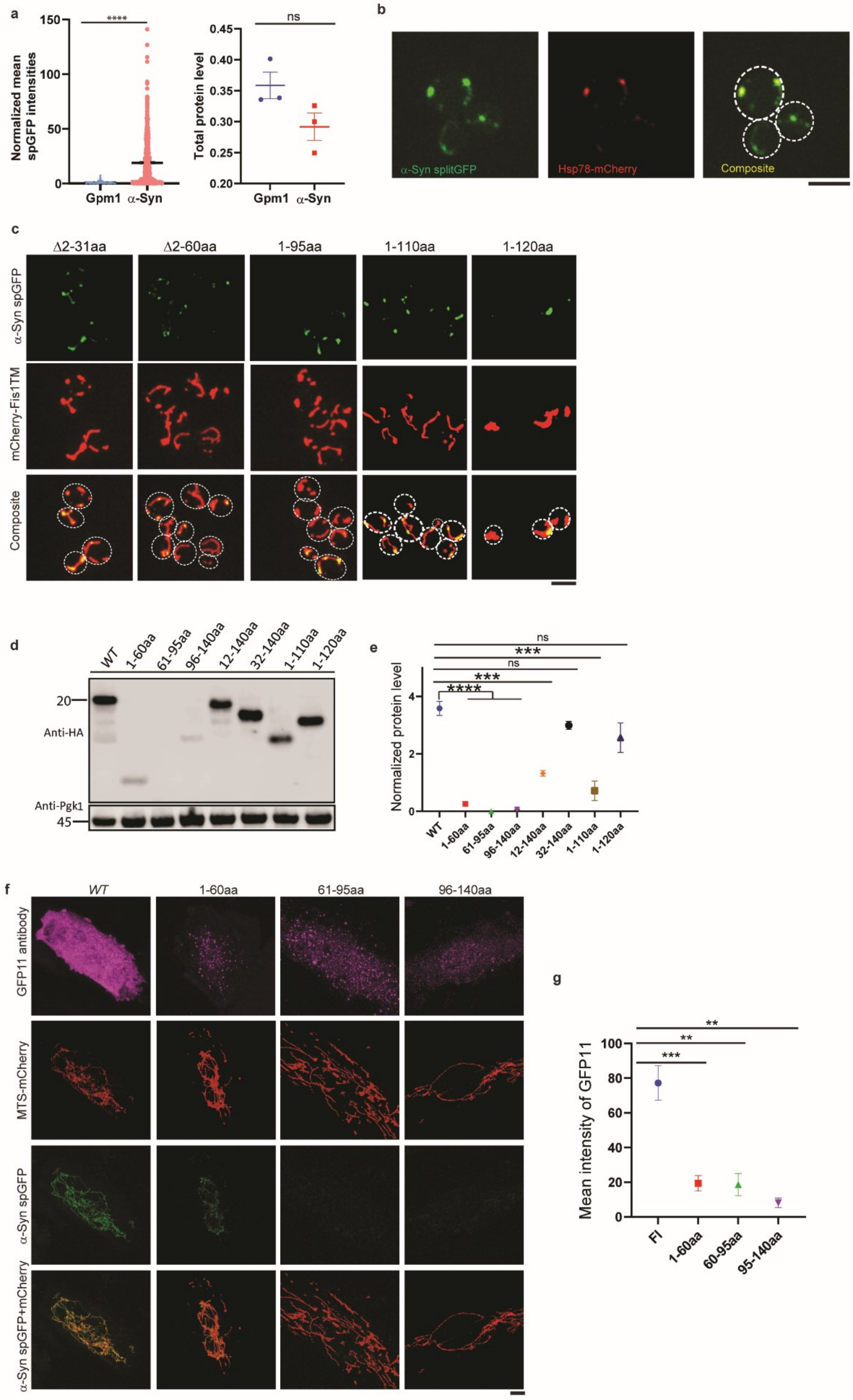
a
Normalized mean spGFP intensities
150
100
50
0
****
Gpm1
α-Syn
Total protein level
0.45
0.40
0.35
0.30
0.25
0.20
ns
b
α-Syn splitGFP
Hsp78-mCherry
Composite
c
Δ2-31aa
Δ2-60aa
1-95aa
1-110aa
1-120aa
α-Syn spGFP
mCherry-Fis1TM
Composite
d
WT
1-60aa
61-95aa
96-140aa
12-140aa
32-140aa
1-110aa
1-120aa
20
Anti-HA
Anti-Pgk1
45
e
Normalized protein level
4
2
0
ns
***
ns
***
****
f
WT
1-60aa
61-95aa
96-140aa
GFP11 antibody
MTS-mCherry
α-Syn spGFP
α-Syn spGFP+mCherry
g
Mean intensity of GFP11
100
80
60
40
20
0
**
**
***
FI
1-60aa
60-95aa
95-140aa

Supplementary Figure 1: Additional data demonstrating α-Syn is imported into mitochondria in yeast and human cells.

a Quantification of mean spGFP intensity acquired by live cell imaging and total protein level by immunoblots of lysates of GFP_{11} labeled α-Syn or Gpm1 yeast strains. Means with individual values or Means ± SEM are shown from 3 biological repeats with unpaired two-tailed *t*-test. **b** Confocal live cell imaging of yeast cells expressing α-Syn spGFP and mCherry labeled Hsp78. Scale bars, 5 μm. **c** Confocal live cell imaging of yeast cells expressing Cherry-Fis1TM, Grx5-$GFP_{1-10,}$ and truncated forms of α-Syn as indicated linking with GFP_{11}. Cell outlines were circled with dashed line. Scale bars, 5 μm. **d** Representative immunoblots of spGFP labeled α-Syn *WT* and mutants in yeast cells as indicated in the figure. HA tag was added between α-Syn and GFP_{11}. **e** Representative immunoblots quantification of spGFP labeled α-Syn *WT* and mutants in yeast cells as indicated in the figure. Means ± SEM are shown from 3 biological repeats with unpaired two-tailed *t*-test between α-Syn *WT* and mutants. **f** Confocal imaging of RPE1 cells expressing MTS-mCherry-GFP_{1-10} and different α-Syn truncations labeled with GFP_{11}. The GFP_{11} antibody was applied for immunostaining of different α-Syn truncations. Scale bars, 5 μm. **g** Quantification of protein levels of the full-length (Fl) and different truncations of Syn-GFP_{11} in RPE1 cells. Shown are Means ± SEM from 3 biological repeat. Unpaired two-tailed *t*-test between α-Syn full-length and truncations as indicated in the figure.

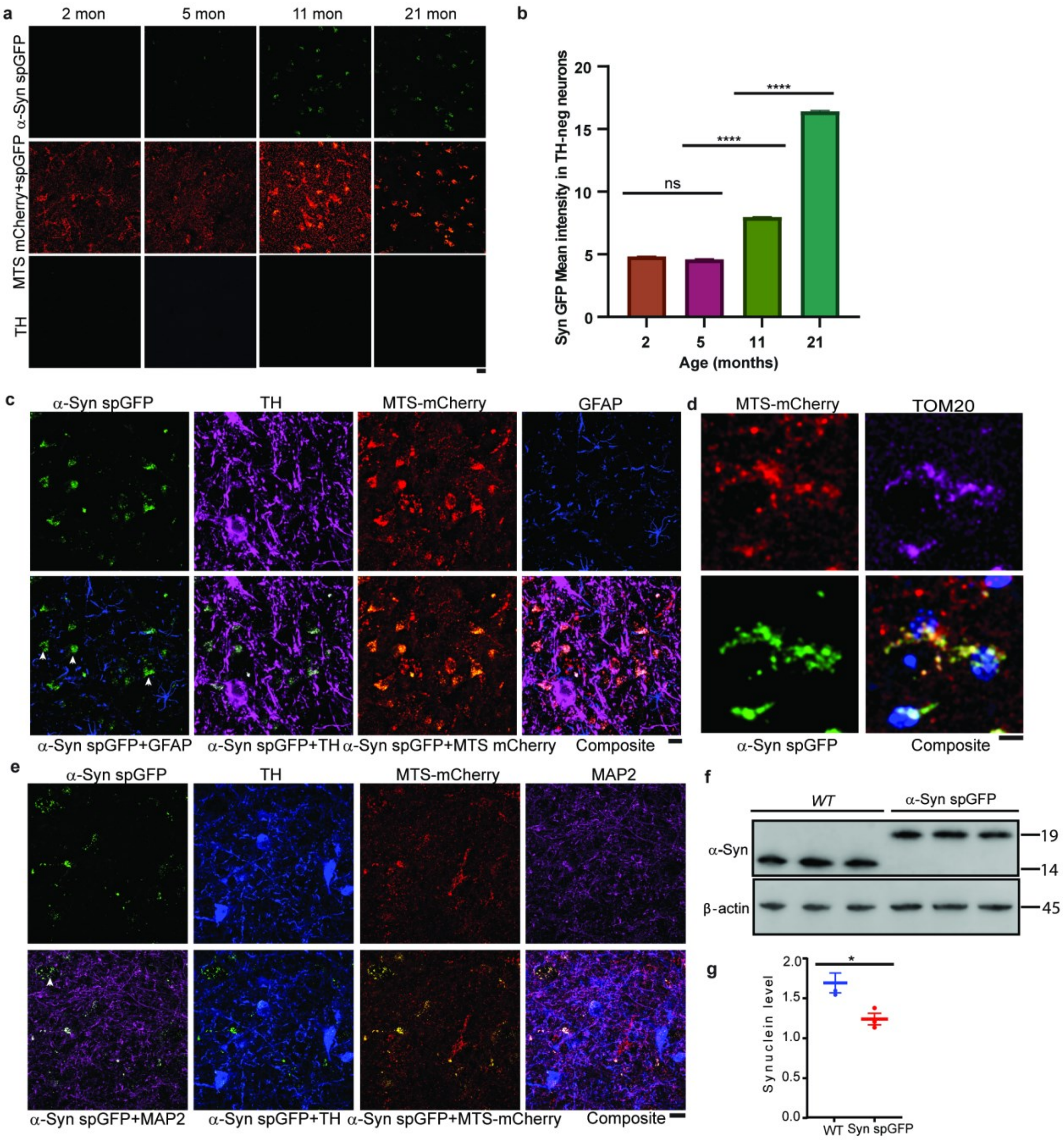

Supplementary Figure 2: α-Syn import into mitochondria is age-dependent and cell type-specific in mouse.

a Representative confocal images of mouse brain slices containing TH-negative cells of 2-, 5-, 11- and 21-months old, comparing with Fig. 2b and 2c. Scale bars, 10 μm. **b** Quantification of the mean intensity of α-Syn spGFP signal in TH-negative cells. *****P* < 0.0001; NS, non-significant;

one-way ANOVA followed by Tukey's multiple comparison test. (*n* = 3-4 brain sections) **c** Representative confocal images of 11 months old mouse brain containing GFAP-positive astrocytes and TH-positive neurons. Scale bars, 10 μm. **d** Representative confocal images of 11 months old mouse brain slice stained with TOM20 antibody and DAPI. Scale bars, 10 μm. **e** Representative confocal images of 11 months old mouse brain containing MAP2-positive neurons. Scale bars, 10 μm. **f** Immunoblots of *WT* and α-Syn spGFP mice midbrain lysis. **g** Quantification of the α-Syn level in the midbrain of *WT* and α-Syn spGFP mice. Shown are Means ± SEM of α-Syn level in midbrain from 3 biological repeats. *P =0.0194; two-way ANOVA test.

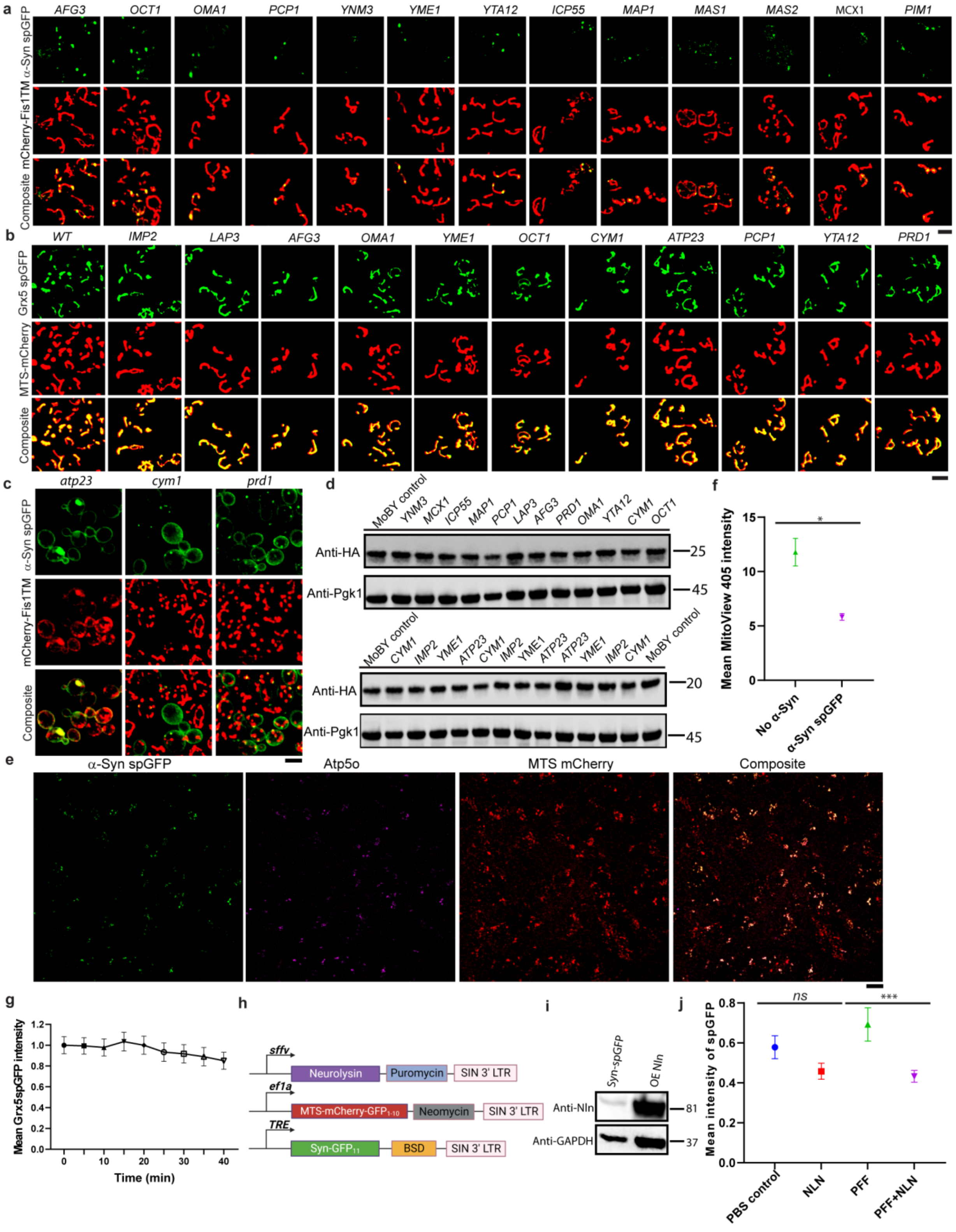
a
AFG3 OCT1 OMA1 PCP1 YNM3 YME1 YTA12 ICP55 MAP1 MAS1 MAS2 MCX1 PIM1
α-Syn spGFP
mCherry-Fis1TM
Composite
b
WT IMP2 LAP3 AFG3 OMA1 YME1 OCT1 CYM1 ATP23 PCP1 YTA12 PRD1
Grx5 spGFP
MTS-mCherry
Composite
c
atp23 cym1 prd1
α-Syn spGFP
mCherry-Fis1TM
Composite
d
MoBY control YNM3 MCX1 ICP55 MAP1 PCP1 LAP3 AFG3 PRD1 OMA1 YTA12 CYM1 OCT1
Anti-HA
25
Anti-Pgk1
45
MoBY control CYM1 IMP2 YME1 ATP23 CYM1 IMP2 YME1 ATP23 ATP23 YME1 IMP2 CYM1 MoBY control
Anti-HA
20
Anti-Pgk1
45
f
Mean MitoView 405 intensity
*
No α-Syn
α-Syn spGFP
e
α-Syn spGFP
Atp5o
MTS mCherry
Composite
g
Mean Grx5spGFP intensity
Time (min)
h
sffv
Neurolysin
Puromycin
SIN 3' LTR
ef1a
MTS-mCherry-GFP1-10
Neomycin
SIN 3' LTR
TRE
Syn-GFP11
BSD
SIN 3' LTR
i
Syn-spGFP
OE Nln
Anti-Nln
81
Anti-GAPDH
37
j
Mean intensity of spGFP
ns
***
PBS control
NLN
PFF
PFF+NLN

Supplementary Figure 3: Additional data demonstrating imported α-Syn is degraded in mitochondria.

a Representative confocal images of cells expressing the α-Syn spGFP system that were introduced with additional copies of individual mitochondrial proteases (as indicated in the figure) via the MoBY plasmid. Scale bars, 5 μm. **b** Representative confocal images of cells expressing the Grx5 spGFP system that were introduced additional copies of individual mitochondrial proteases (as indicated in the figure) via the MoBY plasmid. Scale bars, 5 μm. **c** Representative confocal images of cells expressing the α-Syn spGFP with deletion of *ATP23, CYM1 or PRD1*. Scale bar, 5 μm. **d** Representative immunoblots of α-Syn spGFP that were introduced additional copies of individual mitochondrial proteases via the MoBY plasmid. **e** Confocal images of brain sections of 11-month-old α-Syn spGFP transgenic mice stained with ATP5O antibody. Scale bars, 10 μm. **f** Quantification of the mean MitoView 405 dye intensities in RPE1 cells expressed MTS-mCherry-GFP_{1-10} without (left) or with α-Syn GFP_{11} (right). Shown are Means ± SEM from 3-4 biological repeats with unpaired two-tailed *t*-test. Each data point represents mean dye intensity per biological repeat. **g** Quantification of the mean Grx5 spGFP intensity in time lapse movie of log-phase Grx5 spGFP expressing cells with CCCP added at T0. The interval time is 5 min. Shown are Means ± SEM of Grx5 spGFP intensity in mitochondria from 3 biological repeats. **h** Schematic illustration of the stable RPE1 cell line expressing of α-Syn GFP_{11} under *TRE* promotor and MTS-mCherry-GFP_{1-10} under EF-1α promotor, with or without expression of NLN by lentiviruses transduction. **i** Immunoblots of *WT* and overexpression of Nln in α-Syn spGFP system in RPE1 cells. **j** Quantification of the mean intensity of α-Syn spGFP signal in Fig. 5i. Shown are Means ± SEM of spGFP intensity from 3-5 biological repeats with unpaired two-tailed *t*-test.

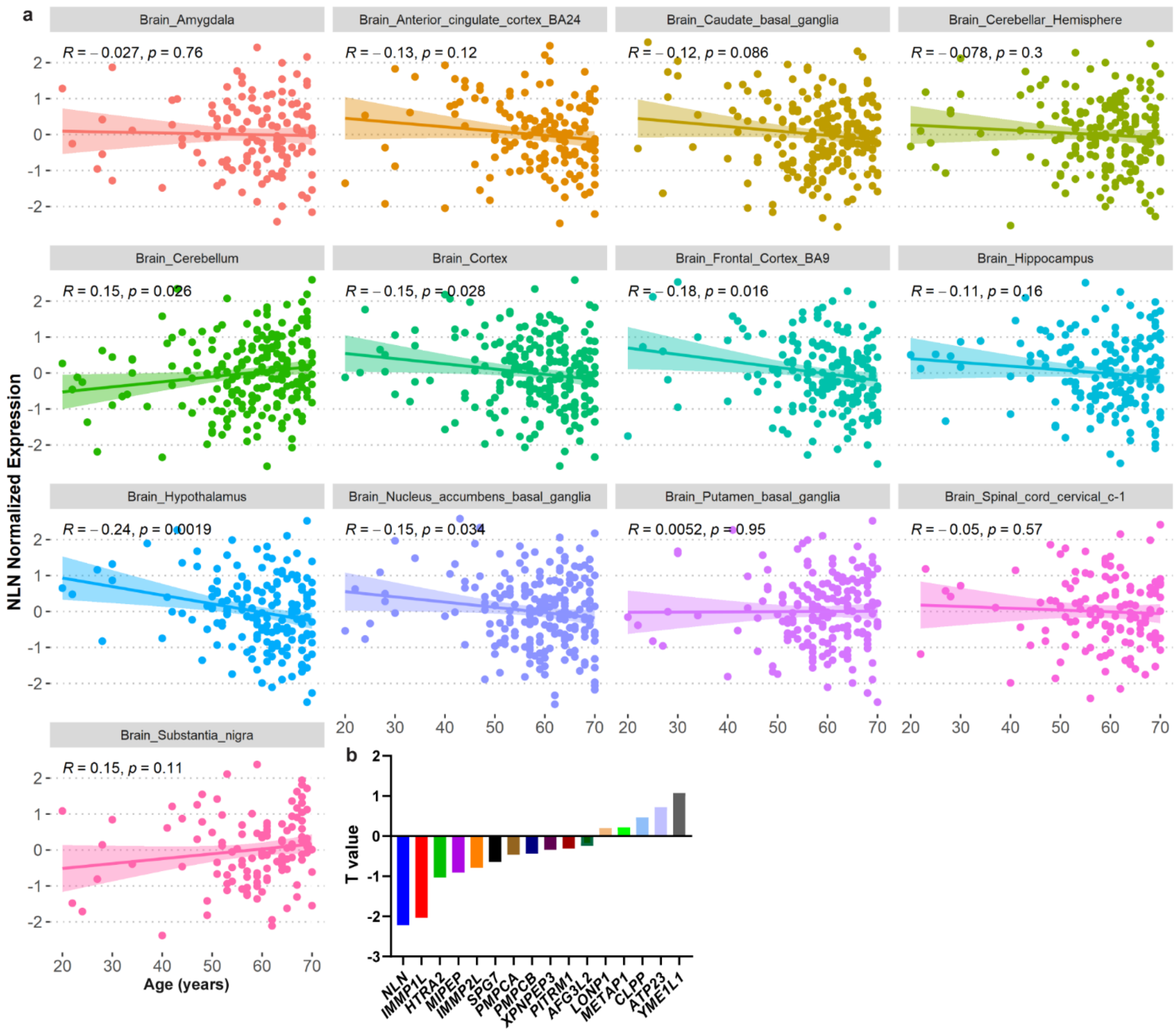

Supplementary Figure 4: RNA-seq analysis for expression of mitochondrial proteases in PD and normal aging.

a Quantification of NLN expression during aging in different brain regions from GTEx RNA-seq database. Shown are expression levels of NLN across GTEx brain tissues for each individual at the time of sample donation (represented as age in x-axis). Expression levels are normalized after regressing out known and hidden covariates described in previous paper[49]. **b** Comparison of transcriptional differences of mitochondrial proteases in DA neurons derived from healthy control and PD patients. T value is derived from generalized linear model estimated using BPSC[59].

Supplementary Table 1: List of yeast strains and plasmids

Strains	Genotype	Source
BY4741	*MATa his3Δ1; leu2Δ0; met15Δ0; ura3Δ0*	NA
RLY11000	*GRX5-GFP*$_{1-10}$*-NatMX6*	This study
RLY11001	*trp::pGAP-mCherry-Fis1TM-KanMX6; GRX5-GFP*$_{1-10}$*-NatMX6*	This study
RLY11002	*Δura3::pGAP-a-Syn-HA-GFP*$_{11}$*-His3MX6; trp::pGAP-mCherry-Fis1TM-KanMX6; GRX5-GFP*$_{1-10}$*-NatMX6; Δpdr5::HygMX6*	This study
RLY11003	*trp::pGAP-mCherry-Fis1TM-KanMX6; GRX5-GFP*$_{1-10}$*-NatMX6; MDH1-HA-GFP*$_{11}$*-His3MX6*	This study
RLY11004	*trp::pGAP-mCherry-Fis1TM-KanMX6; GRX5-GFP*$_{1-10}$*-NatMX6; GPM1-HA-GFP*$_{11}$*-His3MX6*	This study
RLY11005	*Δura3::pGAP-a-Syn-HA-GFP*$_{11}$*-His3MX6; HSP78-mCherry-Ura3MX6; GRX5-GFP*$_{1-10}$*-NatMX6*	This study
RLY11006	*Δura3::pGAP-a-Syn-A53T-HA-GFP*$_{11}$*-His3MX6; trp::pGAP-mCherry-Fis1TM-KanMX6; GRX5-GFP*$_{1-10}$*-NatMX6*	This study
RLY11007	*Δura3::pGAP-a-Syn-A30P-HA-GFP*$_{11}$*-His3MX6; trp::pGAP-mCherry-Fis1TM-KanMX6; GRX5-GFP*$_{1-10}$*-NatMX6*	This study
RLY11008	*Δura3::pGAP-a-Syn-E46K-HA-GFP*$_{11}$*-His3MX6; trp::pGAP-mCherry-Fis1TM-KanMX6; GRX5-GFP*$_{1-10}$*-NatMX6*	This study
RLY11009	*Δura3::pGAP-a-Syn-H50K-HA-GFP*$_{11}$*-His3MX6; trp::pGAP-mCherry-Fis1TM-KanMX6; GRX5-GFP*$_{1-10}$*-NatMX6*	This study
RLY11010	*Δura3::pGAP-a-Syn-1-60aa-HA-GFP*$_{11}$*-His3MX6; trp::pGAP-mCherry-Fis1TM-KanMX6; GRX5-GFP*$_{1-10}$*-NatMX6*	This study
RLY11011	*Δura3::pGAP-a-Syn-61-95aa-HA-GFP*$_{11}$*-His3MX6; trp::pGAP-mCherry-Fis1TM-KanMX6; GRX5-GFP*$_{1-10}$*-NatMX6*	This study
RLY11012	*Δura3::pGAP-a-Syn-61-140aa-HA-GFP*$_{11}$*-His3MX6; trp::pGAP-mCherry-Fis1TM-KanMX6; GRX5-GFP*$_{1-10}$*-NatMX6*	This study
RLY11013	*Δura3::pGAP-a-Syn-96-140aa-HA-GFP*$_{11}$*-His3MX6; trp::pGAP-mCherry-Fis1TM-KanMX6; GRX5-GFP*$_{1-10}$*-NatMX6*	This study
RLY11014	*Δura3::pGAP-a-Syn-32-140aa-HA-GFP*$_{11}$*-His3MX6; trp::pGAP-mCherry-Fis1TM-KanMX6; GRX5-GFP*$_{1-10}$*-NatMX6*	This study
RLY11015	*Δura3::pGAP-a-Syn-1-95aa-HA-GFP*$_{11}$*-His3MX6; trp::pGAP-mCherry-Fis1TM-KanMX6; GRX5-GFP*$_{1-10}$*-NatMX6*	This study
RLY11016	*Δura3::pGAP-a-Syn-1-110aa-HA-GFP*$_{11}$*-His3MX6; trp::pGAP-mCherry-Fis1TM-KanMX6; GRX5-GFP*$_{1-10}$*-NatMX6*	This study
RLY11017	*Δura3::pGAP-a-Syn-1-120aa-HA-GFP*$_{11}$*-His3MX6; trp::pGAP-mCherry-Fis1TM-KanMX6; GRX5-GFP*$_{1-10}$*-NatMX6*	This study
RLY11018	*Δura3::pGAP-a-Syn-HA-GFP*$_{11}$*-His3MX6; trp::pGAP-mCherry-Fis1TM-KanMX6; GRX5-GFP*$_{1-10}$*-NatMX6; Δhsp104::HygMX6*	This study
RLY11019	*Δura3::pGAP-a-Syn-1-60aa-HA-GFP*$_{11}$*-His3MX6; trp::pGAP-mCherry-Fis1TM-KanMX6; GRX5-GFP*$_{1-10}$*-NatMX6; Δhsp104::HygMX6*	This study

RLY11020	*Δura3::pGAP-a-Syn-60-95aa-HA-GFP$_{11}$-His3MX6; trp::pGAP-mCherry-Fis1TM-KanMX6; GRX5-GFP$_{1-10}$-NatMX6; Δhsp104::HygMX6*	This study
RLY11021	*Δura3::pGAP-a-Syn-96-140aa-HA-GFP$_{11}$-His3MX6; trp::pGAP-mCherry-Fis1TM-KanMX6; GRX5-GFP$_{1-10}$-NatMX6; Δhsp104::HygMX6*	This study
RLY11022	*Δura3::pGAP-a-Syn-6xHis-His3MX6*	This study
RLY11023	*trp::MTS-mCherry-6xHis-NatMX6*	This study
RLY11024	*ATP5-mScarlet-hph*	Meurer et al., 2018, Nature Methods
RLY11025	*RLY11024 + Δura3::pGAP-a-Syn-HA-GFP$_{11}$-His3MX6; GRX5-GFP$_{1-10}$-NatMX6*	This study
RLY11026	*NDI1-mScarlet-hph*	Meurer et al., 2018, Nature Methods
RLY11027	*RLY11026 + Δura3::pGAP-a-Syn-HA-GFP11-His3MX6; GRX5-GFP1-10-NatMX6*	This study
RLY11028	*Δura3::pCUP1-a-Syn-HA-GFP$_{11}$-His3MX6; trp::pGAP-mCherry-Fis1TM-KanMX6; GRX5-GFP$_{1-10}$-NatMX6*	This study
RLY11029	*GRX5-GFP$_{11}$-His3MX6; trp1::pGAP-MTS-mCherry-GFP$_{1-10}$-NatMX6; Δpdr5::HygMX6*	This study
RLY11030	*cen::CYM1-Ura3MX6-KanMX6; Δura3::pGAP-a-Syn-HA-GFP$_{11}$-His3MX6; trp::pGAP-mCherry-Fis1TM-KanMX6; GRX5-GFP$_{1-10}$-NatMX6; Δpdr5::HygMX6*	This study
RLY11031	*cen::PRD1-Ura3MX6-KanMX6; Δura3::pGAP-a-Syn-HA-GFP$_{11}$-His3MX6; trp::pGAP-mCherry-Fis1TM-KanMX6; GRX5-GFP$_{1-10}$-NatMX6; Δpdr5::HygMX6*	This study
RLY11032	*cen::ATP23-Ura3MX6-KanMX6; Δura3::pGAP-a-Syn-HA-GFP$_{11}$-His3MX6; trp::pGAP-mCherry-Fis1TM-KanMX6; GRX5-GFP$_{1-10}$-NatMX6; Δpdr5::HygMX6*	This study
RLY11033	*cen::IMP2-Ura3MX6-KanMX6; Δura3::pGAP-a-Syn-HA-GFP$_{11}$-His3MX6; trp::pGAP-mCherry-Fis1TM-KanMX6; GRX5-GFP$_{1-10}$-NatMX6; Δpdr5::HygMX6*	This study
RLY11034	*cen::YME1-Ura3MX6-KanMX6; Δura3::pGAP-a-Syn-HA-GFP$_{11}$-His3MX6; trp::pGAP-mCherry-Fis1TM-KanMX6; GRX5-GFP$_{1-10}$-NatMX6; Δpdr5::HygMX6*	This study
RLY11035	*cen::PCP1-Ura3MX6-KanMX6; Δura3::pGAP-a-Syn-HA-GFP$_{11}$-His3MX6; trp::pGAP-mCherry-Fis1TM-KanMX6; GRX5-GFP$_{1-10}$-NatMX6; Δpdr5::HygMX6*	This study
RLY11036	*cen::MAP1-Ura3MX6-KanMX6; Δura3::pGAP-a-Syn-HA-GFP$_{11}$-His3MX6; trp::pGAP-mCherry-Fis1TM-KanMX6; GRX5-GFP$_{1-10}$-NatMX6; Δpdr5::HygMX6*	This study

RLY11037	*cen::YTA12-Ura3MX6-KanMX6; Δura3::pGAP-a-Syn-HA-GFP_{11}-His3MX6; trp::pGAP-mCherry-Fis1TM-KanMX6; GRX5-GFP_{1-10}-NatMX6; Δpdr5::HygMX6*	This study
RLY11038	*cen::AFG3-Ura3MX6-KanMX6; Δura3::pGAP-a-Syn-HA-GFP_{11}-His3MX6; trp::pGAP-mCherry-Fis1TM-KanMX6; GRX5-GFP_{1-10}-NatMX6; Δpdr5::HygMX6*	This study
RLY11039	*cen::PIM1-Ura3MX6-KanMX6; Δura3::pGAP-a-Syn-HA-GFP_{11}-His3MX6; trp::pGAP-mCherry-Fis1TM-KanMX6; GRX5-GFP_{1-10}-NatMX6; Δpdr5::HygMX6*	This study
RLY11040	*cen::OMA1-Ura3MX6-KanMX6; Δura3::pGAP-a-Syn-HA-GFP_{11}-His3MX6; trp::pGAP-mCherry-Fis1TM-KanMX6; GRX5-GFP_{1-10}-NatMX6; Δpdr5::HygMX6*	This study
RLY11041	*cen::LAP3-Ura3MX6-KanMX6; Δura3::pGAP-a-Syn-HA-GFP_{11}-His3MX6; trp::pGAP-mCherry-Fis1TM-KanMX6; GRX5-GFP_{1-10}-NatMX6; Δpdr5::HygMX6*	This study
RLY11042	*cen::ICP55-Ura3MX6-KanMX6; Δura3::pGAP-a-Syn-HA-GFP_{11}-His3MX6; trp::pGAP-mCherry-Fis1TM-KanMX6; GRX5-GFP_{1-10}-NatMX6; Δpdr5::HygMX6*	This study
RLY11043	*cen::YNM3-Ura3MX6-KanMX6; Δura3::pGAP-a-Syn-HA-GFP_{11}-His3MX6; trp::pGAP-mCherry-Fis1TM-KanMX6; GRX5-GFP_{1-10}-NatMX6; Δpdr5::HygMX6*	This study
RLY11044	*cen::OCT1-Ura3MX6-KanMX6; Δura3::pGAP-a-Syn-HA-GFP_{11}-His3MX6; trp::pGAP-mCherry-Fis1TM-KanMX6; GRX5-GFP_{1-10}-NatMX6; Δpdr5::HygMX6*	This study
RLY11045	*cen::MCX1-Ura3MX6-KanMX6; Δura3::pGAP-a-Syn-HA-GFP_{11}-His3MX6; trp::pGAP-mCherry-Fis1TM-KanMX6; GRX5-GFP_{1-10}-NatMX6; Δpdr5::HygMX6*	This study
RLY11046	*cen::MAS1-Ura3MX6-KanMX6; Δura3::pGAP-a-Syn-HA-GFP_{11}-His3MX6; trp::pGAP-mCherry-Fis1TM-KanMX6; GRX5-GFP_{1-10}-NatMX6; Δpdr5::HygMX6*	This study
RLY11047	*cen::MAS2-Ura3MX6-KanMX6; Δura3::pGAP-a-Syn-HA-GFP_{11}-His3MX6; trp::pGAP-mCherry-Fis1TM-KanMX6; GRX5-GFP_{1-10}-NatMX6; Δpdr5::HygMX6*	This study
RLY11048	*cen::CYM1-Ura3MX6-KanMX6;GRX5-GFP_{11}-His3MX6; trp1::pGAP-MTS-mCherry-GFP_{1-10}-NatMX6*	This study
RLY11049	*cen::PRD1-Ura3MX6-KanMX6;GRX5-GFP_{11}-His3MX6; trp1::pGAP-MTS-mCherry-GFP_{1-10}-NatMX6*	This study
RLY11050	*cen::ATP23-Ura3MX6-KanMX6;GRX5-GFP_{11}-His3MX6; trp1::pGAP-MTS-mCherry-GFP_{1-10}-NatMX6*	This study
RLY11051	*cen::IMP2-Ura3MX6-KanMX6;GRX5-GFP_{11}-His3MX6; trp1::pGAP-MTS-mCherry-GFP_{1-10}-NatMX6*	This study
RLY11052	*cen::YME1-Ura3MX6-KanMX6;GRX5-GFP_{11}-His3MX6; trp1::pGAP-MTS-mCherry-GFP_{1-10}-NatMX6*	This study
RLY11053	*cen::PCP1-Ura3MX6-KanMX6;GRX5-GFP_{11}-His3MX6; trp1::pGAP-MTS-mCherry-GFP_{1-10}-NatMX6*	This study

RLY11054	cen::MAP1-Ura3MX6-KanMX6;GRX5-GFP_{11}-His3MX6; trp1::pGAP-MTS-mCherry-GFP_{1-10}-NatMX6	This study
RLY11055	cen::YTA12-Ura3MX6-KanMX6;GRX5-GFP_{11}-His3MX6; trp1::pGAP-MTS-mCherry-GFP_{1-10}-NatMX6	This study
RLY11056	cen::AFG3-Ura3MX6-KanMX6;GRX5-GFP_{11}-His3MX6; trp1::pGAP-MTS-mCherry-GFP_{1-10}-NatMX6	This study
RLY11057	cen::PIM1-Ura3MX6-KanMX6;GRX5-GFP_{11}-His3MX6; trp1::pGAP-MTS-mCherry-GFP_{1-10}-NatMX6	This study
RLY11058	cen::OMA1-Ura3MX6-KanMX6;GRX5-GFP_{11}-His3MX6; trp1::pGAP-MTS-mCherry-GFP_{1-10}-NatMX6	This study
RLY11059	cen::LAP3-Ura3MX6-KanMX6;GRX5-GFP_{11}-His3MX6; trp1::pGAP-MTS-mCherry-GFP_{1-10}-NatMX6	This study
RLY11060	cen::ICP55-Ura3MX6-KanMX6;GRX5-GFP_{11}-His3MX6; trp1::pGAP-MTS-mCherry-GFP_{1-10}-NatMX6	This study
RLY11061	cen::YNM3-Ura3MX6-KanMX6;GRX5-GFP_{11}-His3MX6; trp1::pGAP-MTS-mCherry-GFP_{1-10}-NatMX6	This study
RLY11062	cen::OCT1-Ura3MX6-KanMX6;GRX5-GFP_{11}-His3MX6; trp1::pGAP-MTS-mCherry-GFP_{1-10}-NatMX6	This study
RLY11063	cen::MCX1-Ura3MX6-KanMX6;GRX5-GFP_{11}-His3MX6; trp1::pGAP-MTS-mCherry-GFP_{1-10}-NatMX6	This study
RLY11064	cen::MAS1-Ura3MX6-KanMX6;GRX5-GFP_{11}-His3MX6; trp1::pGAP-MTS-mCherry-GFP_{1-10}-NatMX6	This study
RLY11065	cen::MAS2-Ura3MX6-KanMX6;GRX5-GFP_{11}-His3MX6; trp1::pGAP-MTS-mCherry-GFP_{1-10}-NatMX6	This study
RLY11066	Δura3::pGAP-a-Syn-HA-GFP_{11}-His3MX6; trp::pGAP-mCherry-Fis1TM-KanMX6; GRX5-GFP_{1-10}-NatMX6; Δatp23::HygMX6	This study
RLY11067	Δura3::pGAP-a-Syn-HA-GFP_{11}-His3MX6; trp::pGAP-mCherry-Fis1TM-KanMX6; GRX5-GFP_{1-10}-NatMX6; Δcym1::HygMX6	This study
RLY11068	Δura3::pGAP-a-Syn-HA-GFP_{11}-His3MX6; trp::pGAP-mCherry-Fis1TM-KanMX6; GRX5-GFP_{1-10}-NatMX6; Δprd1::HygMX6	This study
RLY11069	Δhis3::GEM-pGal-a-Syn-HA-GFP_{11}-His3MX6; trp::pGAP-mCherry-Fis1TM-HygMX6; GRX5-GFP_{1-10}-NatMX6;	This study
RLY11070	Δura3::pGAP-a-Syn-HA-GFP_{11}-KanMX6;Δhis3::GEM-pGal-a-Syn-HA-GFP_{11}-His3MX6; trp::pGAP-mCherry-Fis1TM-HygMX6; GRX5-GFP_{1-10}-NatMX6;	This study
RLY11071	cen::ATP23-Ura3MX6; Δura3::pGAP-a-Syn-HA-GFP_{11}-KanMX6;Δhis3::GEM-pGal-a-Syn-HA-GFP_{11}-His3MX6; trp::pGAP-mCherry-Fis1TM-HygMX6; GRX5-GFP_{1-10}-NatMX6;	This study
RLY11072	cen::CYM1-Ura3MX6; Δura3::pGAP-a-Syn-HA-GFP_{11}-KanMX6;Δhis3::GEM-pGal-a-Syn-HA-GFP_{11}-His3MX6; trp::pGAP-mCherry-Fis1TM-HygMX6; GRX5-GFP_{1-10}-NatMX6;	This study
RLY11073	cen::PRD1-Ura3MX6; Δura3::pGAP-a-Syn-HA-GFP_{11}-KanMX6;Δhis3::GEM-pGal-a-Syn-HA-GFP_{11}-His3MX6; trp::pGAP-mCherry-Fis1TM-HygMX6; GRX5-GFP_{1-10}-NatMX6;	This study

RLY11074	*cen::IMP2-Ura3MX6; Δura3::pGAP-a-Syn-HA-GFP*$_{11}$*-KanMX6;Δhis3::GEM-pGal-a-Syn-HA-GFP*$_{11}$*-His3MX6; trp::pGAP-mCherry-Fis1TM-HygMX6; GRX5-GFP*$_{1\text{-}10}$*-NatMX6;*	This study
RLY11075	*cen::YNM3-Ura3MX6; Δura3::pGAP-a-Syn-HA-GFP*$_{11}$*-KanMX6;Δhis3::GEM-pGal-a-Syn-HA-GFP*$_{11}$*-His3MX6; trp::pGAP-mCherry-Fis1TM-HygMX6; GRX5-GFP*$_{1\text{-}10}$*-NatMX6;*	This study
RLY11076	*cen::PRD1-Ura3MX6; Δura3::pGAP-a-Syn-HA-GFP*$_{11}$*-KanMX6;Δhis3::GEM-pGal-a-Syn-HA-GFP*$_{11}$*-His3MX6; GRX5-GFP*$_{1\text{-}10}$*-NatMX6;*	This study
RLY11077	*cen::YME1-Ura3MX6; Δura3::pGAP-a-Syn-HA-GFP*$_{11}$*-KanMX6;Δhis3::GEM-pGal-a-Syn-HA-GFP*$_{11}$*-His3MX6; GRX5-GFP*$_{1\text{-}10}$*-NatMX6;*	This study
RLY11078	*cen::IMP2-Ura3MX6; Δura3::pGAP-a-Syn-HA-GFP*$_{11}$*-KanMX6;Δhis3::GEM-pGal-a-Syn-HA-GFP*$_{11}$*-His3MX6; GRX5-GFP*$_{1\text{-}10}$*-NatMX6;*	This study
RLY11079	*cen::ATP23-Ura3MX6; Δura3::pGAP-a-Syn-HA-GFP*$_{11}$*-KanMX6;Δhis3::GEM-pGal-a-Syn-HA-GFP*$_{11}$*-His3MX6; GRX5-GFP*$_{1\text{-}10}$*-NatMX6;*	This study
RLY11080	*cen::CYM1-Ura3MX6; Δura3::pGAP-a-Syn-HA-GFP*$_{11}$*-KanMX6;Δhis3::GEM-pGal-a-Syn-HA-GFP*$_{11}$*-His3MX6; GRX5-GFP*$_{1\text{-}10}$*-NatMX6;*	This study

**Plasmid ID**	**Construct**	**Source**	**Vector type**
RLB2000	*TRP1::pGAP-MTS-mCherry*	Ruan, L. et al., 2017, Nature	Yeast expression
RLB2001	*pGAP-a-Syn-HA-GFP*$_{11}$*-His3MX6*	This study	Yeast expression
RLB2002	*pGAP-a-Syn-HA-GFP*$_{11}$*-KanMX6*	This study	Yeast expression
RLB2003	*pCUP1-a-Syn-HA-GFP*$_{11}$*-His3MX6*	This study	Yeast expression
RLB2004	*GEM-pGAL1-a-Syn-HA-GFP*$_{11}$*-His3MX6*	This study	Yeast expression
RLB2005	*pCMV-MTS-mCherry-GFP*$_{1\text{-}10}$	Ruan, L. et al., 2017, Nature	Mammalian expression
RLB2006	*pCMV-a-Syn-GFP*$_{11}$	This study	Mammalian expression
RLB2007	*pCMV-a-Syn-1-60aa-GFP*$_{11}$	This study	Mammalian expression
RLB2008	*pCMV-a-Syn-61-95aa-GFP*$_{11}$	This study	Mammalian expression
RLB2009	*pCMV-a-Syn-96-140aa-GFP*$_{11}$	This study	Mammalian expression
RLB2010	*pCAG-MTS-mCherry-GFP1-10-Rosa 26 arm*	This study	Mammalian expression

RLB2011	*pTRE-a-Syn-GFP$_{11}$-Bsd*	This study	Mammalian expression
RLB2012	*pEF1a-MTS-mCherry-GFP$_{1-10}$-Neo*	This study	Mammalian expression
RLB2013	*pSFFV-NLN-Puro*	This study	Mammalian expression
12260	*psPAX2*	Addgene	Mammalian expression
12259	*pMD2.G*	Addgene	Mammalian expression

Supplementary Video 1: Time-lapse movie showing that acute induction of α-Syn spGFP caused mitochondrial fragmentation in yeast. Mitochondria outer membrane was labeled with Tom70-mCherry. The interval time is 30 min.

Supplementary Video 2: Time-lapse movie recording yeast cell expressing α-Syn spGFP for 40 min. Mitochondria outer membrane was labeled with Tom70-mCherry. The interval time is 5 min.

Supplementary Video 3: Time-lapse movie recording yeast cell expressing α-Syn spGFP that were treated with CCCP for 40 min. Mitochondria outer membrane was labeled with Tom70-mCherry. The interval time is 5 min.

Supplementary Video 4: Time-lapse movie recording yeast cell expressing Grx5 spGFP that were treated with CCCP for 40 min. Mitochondria outer membrane was labeled with Tom70-mCherry. The interval time is 5 min.

Uncropped western blots of Fig. 1e

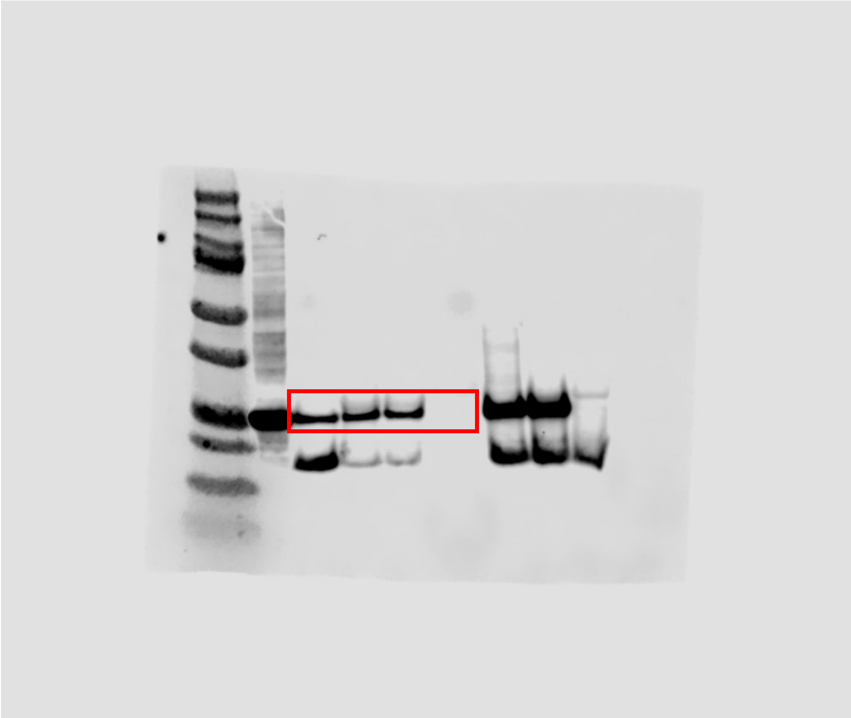

HA Tag antibody (pGAPα-Syn-HA-GFP11-His3MX6)

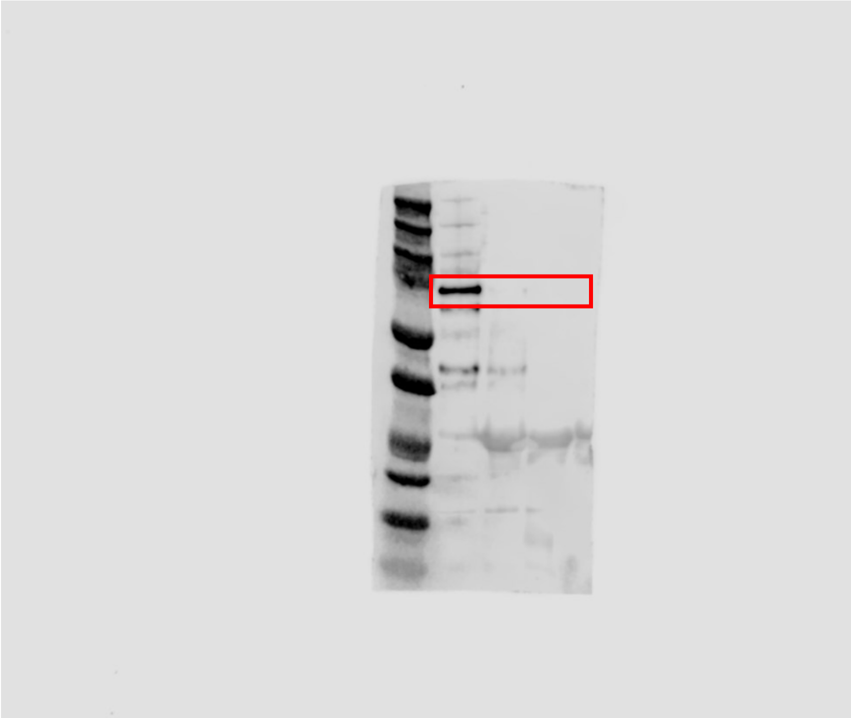

Tom70 antibody

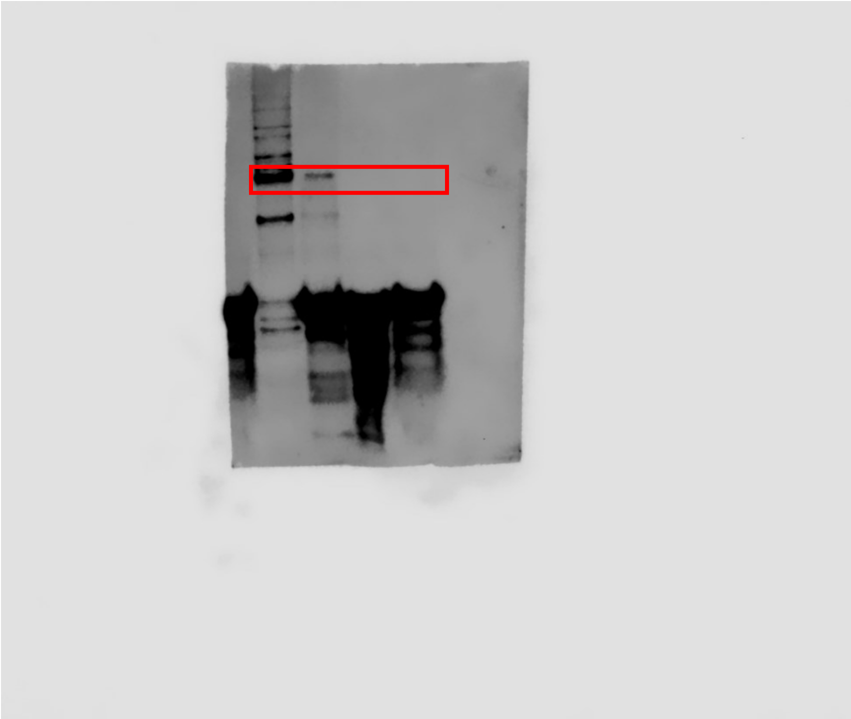

Dld1 antibody

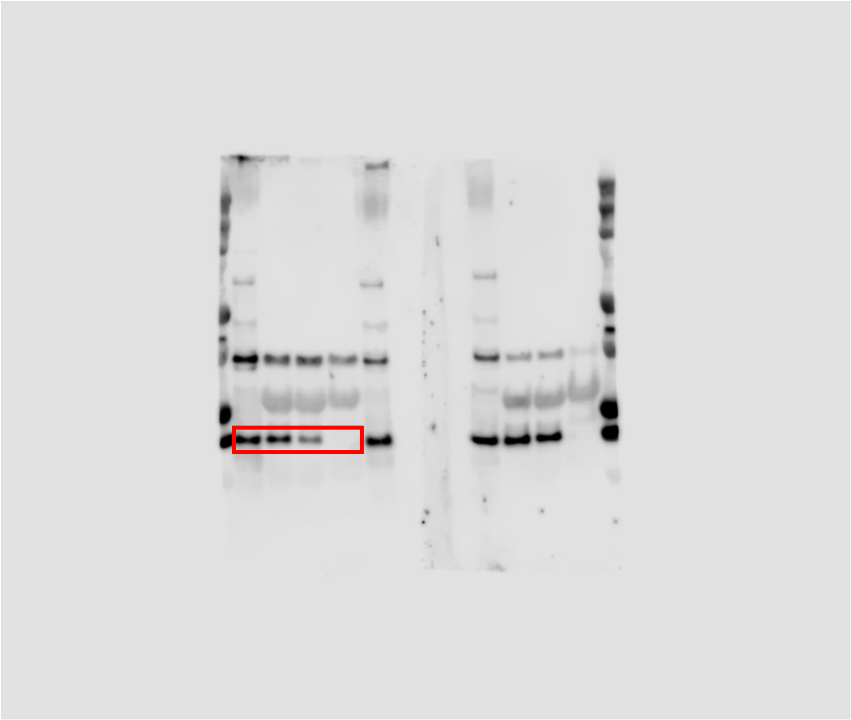

Abf2 antibody.

Red boxes indicate cropped areas shown in the figure.

Uncropped western blots of Supplementary Fig. 1d

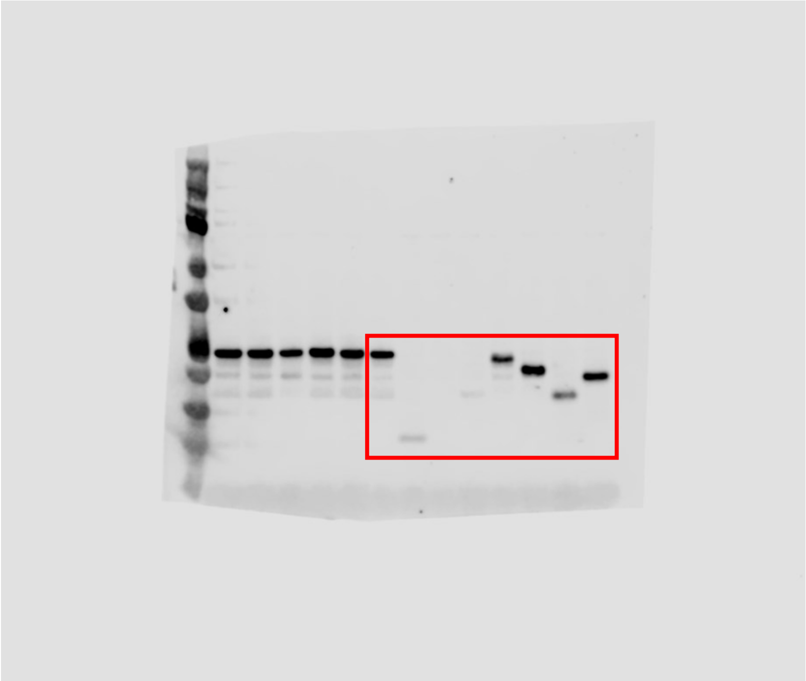

HA Tag antibody

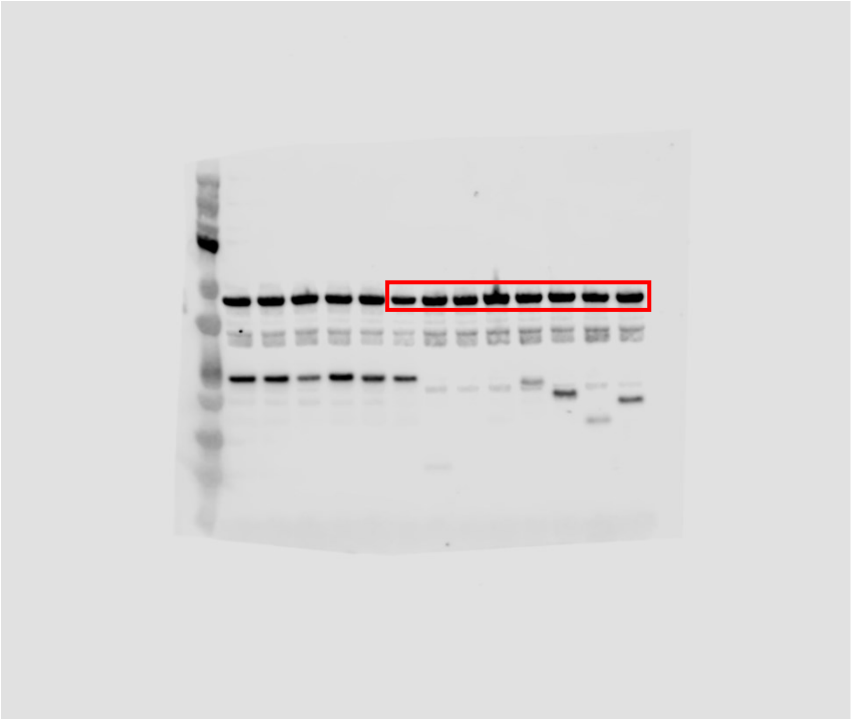

Pgk1 antibody

Red boxes indicate cropped areas shown in the figure.

Uncropped western blots of Supplementary Fig. 2f

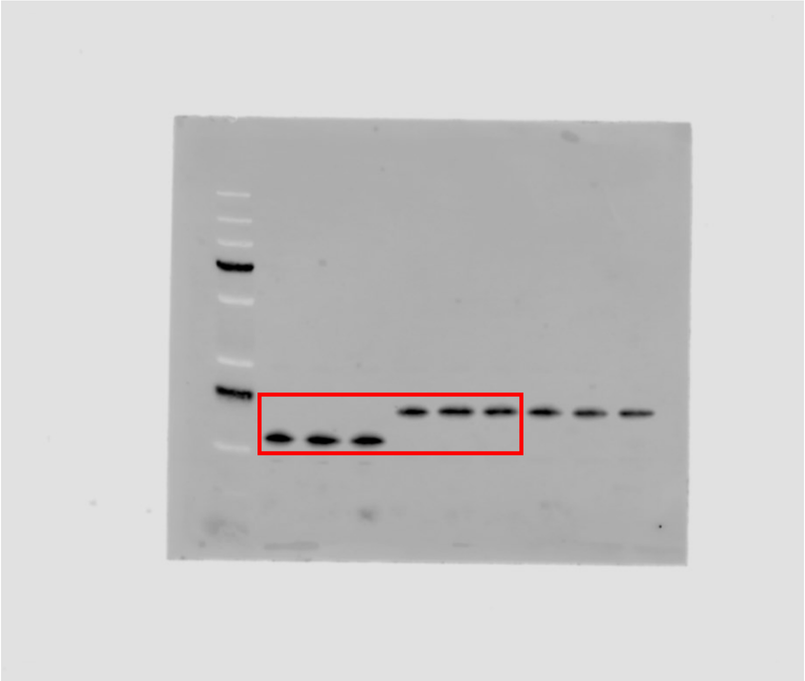

Mouse α-Syn antibody

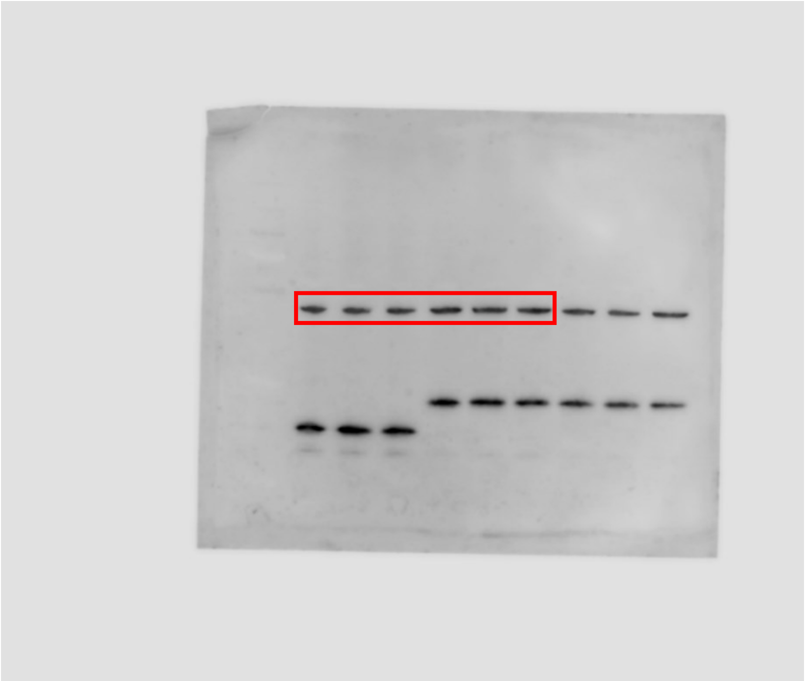

β-actin antibody

Red boxes indicate cropped areas shown in the figure.

Uncropped western blots of Supplementary Fig. 3d

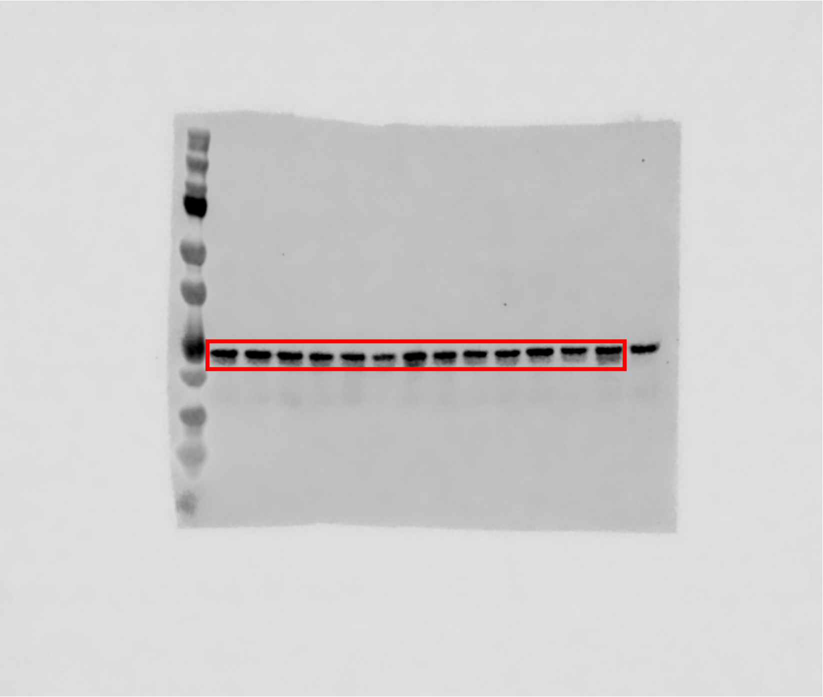

HA Tag antibody

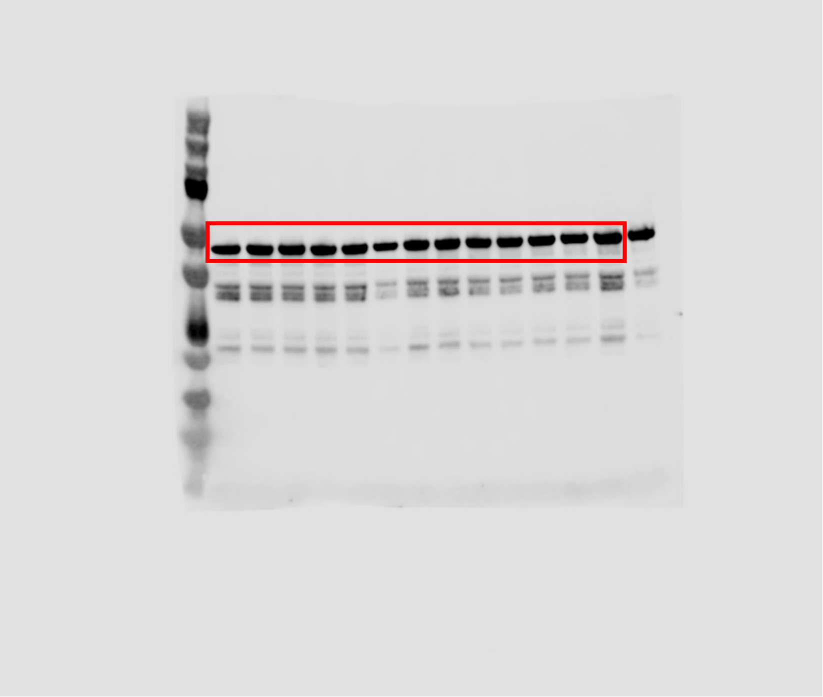

Pgk1 antibody

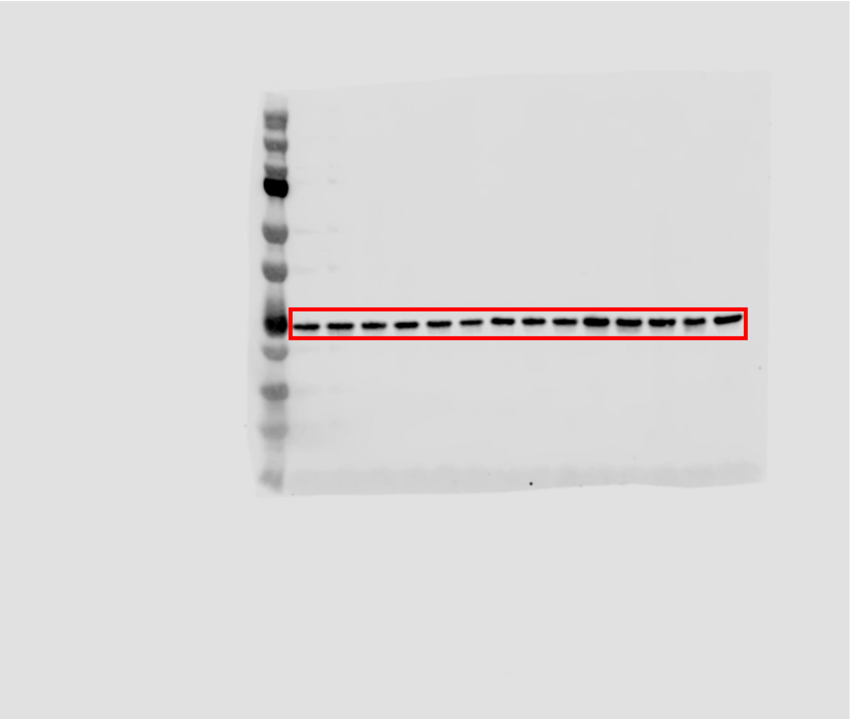

HA Tag antibody

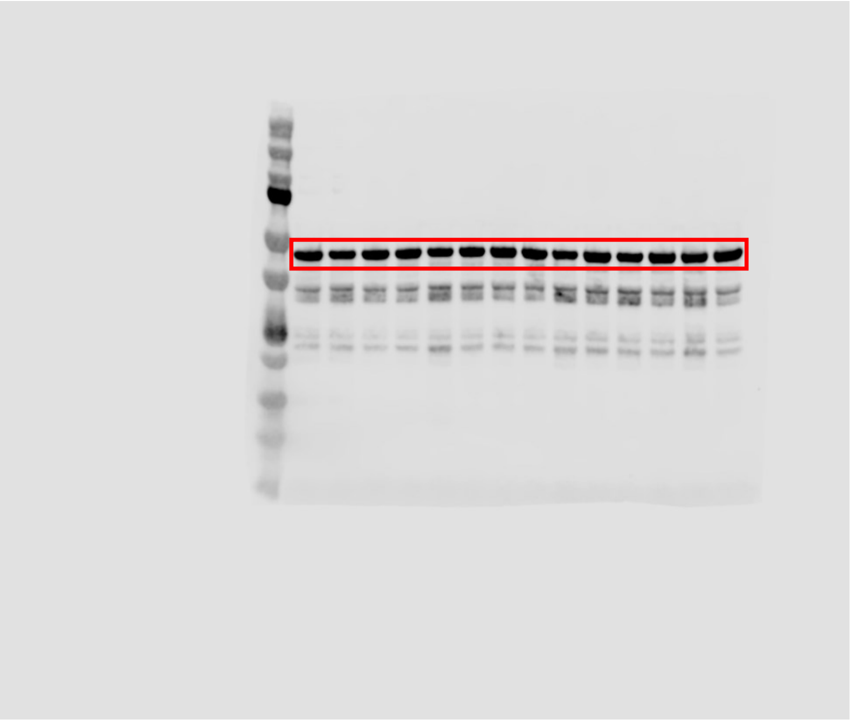

Pgk1 antibody

Red boxes indicate cropped areas shown in the figure.

Uncropped western blots of Supplementary Fig. 3i

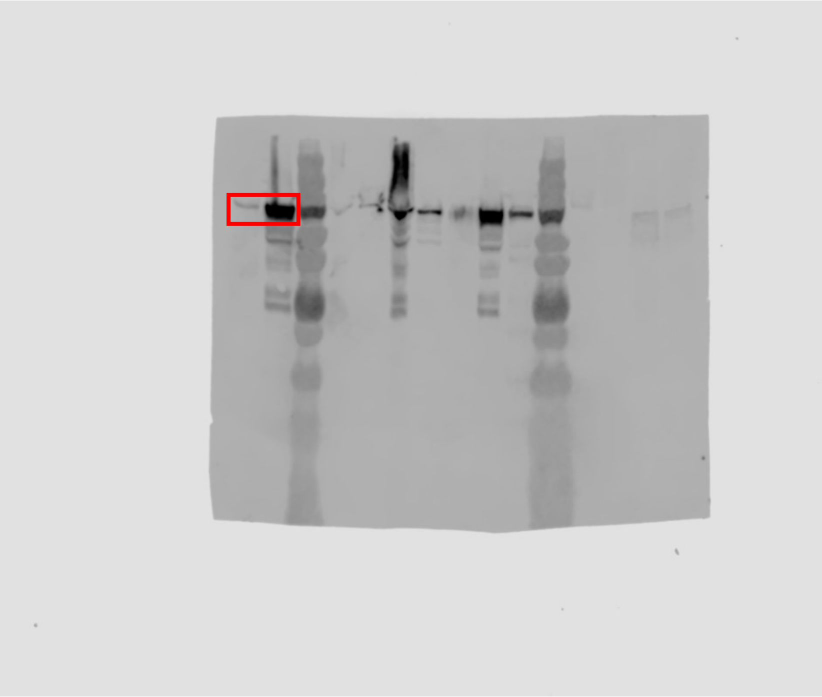

Nln antibody

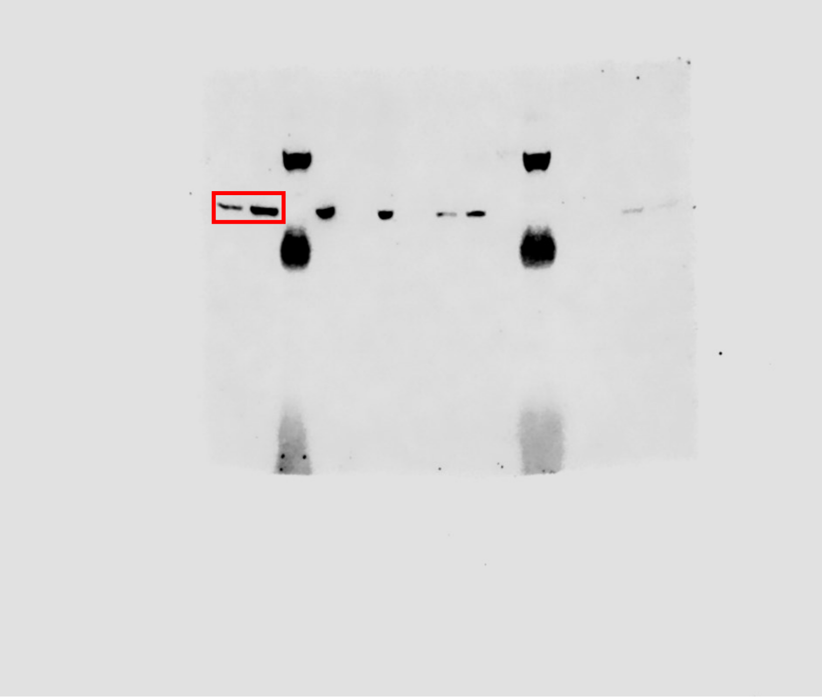

GAPDH antibody

Red boxes indicate cropped areas shown in the figure.
